# External validation of models to predict the outcome of pregnancies of unknown location: a multicentre cohort study

**DOI:** 10.1111/1471-0528.16497

**Published:** 2020-10-07

**Authors:** E Christodoulou, S Bobdiwala, C Kyriacou, J Farren, N Mitchell‐Jones, F Ayim, B Chohan, O Abughazza, B Guruwadahyarhalli, M Al‐Memar, S Guha, V Vathanan, D Gould, C Stalder, L Wynants, D Timmerman, T Bourne, B Van Calster

**Affiliations:** ^1^ Department of Development and Regeneration KU Leuven Leuven Belgium; ^2^ Tommy's National Centre for Miscarriage Research Queen Charlotte's & Chelsea Hospital Imperial College London UK; ^3^ St Marys' Hospital London UK; ^4^ Chelsea and Westminster NHS Trust London UK; ^5^ Hillingdon Hospital London UK; ^6^ Wexham Park Hospital Slough UK; ^7^ Royal Surrey County Hospital Guildford UK; ^8^ Department of Epidemiology CAPHRI Care and Public Health Research Institute Maastricht University Maastricht The Netherlands; ^9^ Department of Obstetrics and Gynaecology University Hospitals Leuven Leuven Belgium; ^10^ Department of Biomedical Data Sciences Leiden University Medical Centre Leiden The Netherlands; ^11^ EPI‐Centre, KU Leuven Leuven Belgium

**Keywords:** Beta human chorionic gonadotrophin ratio, ectopic pregnancy, prediction model, prediction model validation, pregnancy of unknown location, progesterone

## Abstract

**Objective:**

To validate externally five approaches to predict ectopic pregnancy (EP) in pregnancies of unknown location (PUL): the M6P and M6NP risk models, the two‐step triage strategy (2ST, which incorporates M6P), the M4 risk model, and beta human chorionic gonadotropin ratio cut‐offs (BhCG‐RC).

**Design:**

Secondary analysis of a prospective cohort study.

**Setting:**

Eight UK early pregnancy assessment units.

**Population:**

Women presenting with a PUL and BhCG >25 IU/l.

**Methods:**

Women were managed using the 2ST protocol: PUL were classified as low risk of EP if presenting progesterone ≤2 nmol/l; the remaining cases returned 2 days later for triage based on M6P. EP risk ≥5% was used to classify PUL as high risk. Missing values were imputed, and predictions for the five approaches were calculated post hoc. We meta‐analysed centre‐specific results.

**Main outcome measures:**

Discrimination, calibration and clinical utility (decision curve analysis) for predicting EP.

**Results:**

Of 2899 eligible women, the primary analysis excluded 297 (10%) women who were lost to follow up. The area under the ROC curve for EP was 0.89 (95% CI 0.86–0.91) for M6P, 0.88 (0.86–0.90) for 2ST, 0.86 (0.83–0.88) for M6NP and 0.82 (0.78–0.85) for M4. Sensitivities for EP were 96% (M6P), 94% (2ST), 92% (N6NP), 80% (M4) and 58% (BhCG‐RC); false‐positive rates were 35%, 33%, 39%, 24% and 13%. M6P and 2ST had the best clinical utility and good overall calibration, with modest variability between centres.

**Conclusions:**

2ST and M6P performed best for prediction and triage in PUL.

**Tweetable abstract:**

The M6 model, as part of a two‐step triage strategy, is the best approach to characterise and triage PULs.

## Tweetable abstract

The M6 model, as part of a two‐step triage strategy, is the best approach to characterise and triage PULs.

## Introduction

Pregnancy of unknown location (PUL) refers to a situation where a woman has a positive pregnancy test but the pregnancy cannot be definitively located inside or outside the endometrial cavity based on transvaginal sonography. Reported PUL rates vary between 5% and 42% depending on the local setting.^1^ The outcome can be a failing pregnancy (FPUL), an intrauterine pregnancy (IUP), an ectopic pregnancy (EP) or exceptionally a persistent PUL (PPUL).^1^, ^2^ The concern is that women presenting with a PUL may have an unseen EP, with reported rates of 5–20%.^1^ Because EP has higher complication rates than FPUL or IUP, management of PUL should focus on cases with an increased risk of being an EP while by avoiding unnecessary visits and blood tests for other PUL.

A single progesterone level of ≤10 nmol/l has been used to discharge women with PUL after their initial visit because of a high likelihood of FPUL.^3^, ^4^, ^5^ A beta human chorionic gonadotrophin (BhCG) ratio change over 48 hours between a 13% decrease and a 66% increase has been used to identify PUL at high risk of EP. This refers to a ‘BhCG ratio’ (BhCG at 48 hours/BhCG at 0 hours) between 0.87 and 1.66.^6^, ^7^, ^8^ Alternatively, risk prediction models have been used, of which the M4 model is the most well‐known.^9^, ^10^ M4 estimates the probability of a PUL being FPUL, IUP or EP (including PPUL) based on the initial BhCG and BhCG ratio. A recent meta‐analysis suggested that M4 is superior to single progesterone and BhCG ratio to identify PUL at high risk of EP.^11^ However, an external validation study suggested that M4 gives risk estimates for EP that are too low.^10^

Recently, the M6 risk model was developed to improve M4.^12^ M6 also estimates the probability of a PUL being FPUL, IUP or EP. The predictors for M6 are the initial BhCG, BhCG ratio and initial progesterone. The initial progesterone is a recommended yet optional predictor, so M6 can be used either with (M6P) or without (M6NP) it. In addition, a two‐step triage strategy (2ST) was proposed to further rationalise the management of PUL (Figure S1).^12^ Specifically, Step 1 classifies PUL as low risk of EP if the initial progesterone is ≤2 nmol/l. For remaining cases, a second blood sample after 48 hours is recommended to calculate risk based on M6P.

This study aimed to externally validate 2ST, M6P, M6NP, M4 and BhCG ratio cut‐offs to predict EP in PUL.

## Methods

### Design, setting and participants

This study is a secondary analysis of a prospective multicentre clinical study to investigate the safety of the 2ST when used in clinical practice.^13^ Patients presenting with a PUL at their initial visit were consecutively recruited between January 2015 and January 2017 at the dedicated early pregnancy assessment unit of eight hospitals in the UK. Four university teaching hospitals (Queen Charlotte's and Chelsea, St Mary's, Chelsea and Westminster, West Middlesex University Hospital) and four district general hospitals (Hillingdon, North Middlesex, Wexham Park, Royal Surrey) participated.

Women classified as having a PUL after their first transvaginal scan and who were suitable for outpatient management were included. At their initial visit at the early pregnancy assessment unit, women had a transvaginal ultrasound scan performed by a trained sonographer. Women were classified as having a PUL if they had a positive urine pregnancy test and the location of the pregnancy could not be clearly defined on transvaginal sonography.^2^ We excluded haemodynamically unstable women and women who could not be safely managed as an outpatient (e.g. moderate to severe pelvic pain, haemoperitoneum on transvaginal sonography). For this external validation study, we additionally excluded patients presenting with a BhCG level of ≤25 IU/l, as this is the level at which most urine pregnancy tests have a negative result. Further details of study procedures can be found in the report on the primary objective of the study.^13^ The study was registered as an audit and therefore ethical and written consent was not required following consultation with a national research ethics authority as well as local research, development and audit departments. There was no patient or public involvement for this study.

We followed the TRIPOD (Transparent Reporting of a multivariable prediction model for Individual Prognosis or Diagnosis) reporting guidelines.^14^

### Data collection

The primary aim of the study was to evaluate the implementation of the 2ST on clinical outcomes. Data were collected into a protected Microsoft EXCEL spreadsheet (Microsoft Corporation, One Microsoft Way, Redmond, WA, USA). The 2ST was embedded in the spreadsheet, such that clinicians immediately received the Step 1 and Step 2 recommendations when the appropriate data were entered. All women with a PUL had a blood sample taken at the first healthcare visit for measurement of serum BhCG and progesterone levels. The study protocol instructed that a second blood sample 48 hours later (± 8 hours) be scheduled for a second measurement of serum BhCG if the presenting progesterone level was >2 nmol/l (Step 1 of 2ST; Figure S1). If presenting progesterone was ≤2 nmol/l, the PUL was classified as low risk of EP (likely FPUL). In these cases, a clinically important EP would be very unlikely and therefore a second blood sample to determine the BhCG ratio is not needed. One centre (West Middlesex) decided to collect a second sample only when the progesterone was >10 nmol/l. Each hospital used laboratory assays and kits as per local practice, hence these could vary between hospitals. Using the second BhCG level, the EXCEL document automatically calculated the BhCG ratio and estimated the probability of FPUL, IUP and EP using M6P (Step 2 of 2ST). Patients received management recommendations according to the classification given by 2ST, see Appendix S1 and the primary publication of this study.^13^

As the 2ST was implemented in routine clinical practice, deviations were possible. For example, some patients did not have progesterone measured at presentation (e.g. if this is not local routine practice), or were on progesterone supplements. In these cases, the spreadsheet automatically used M6NP to estimate the probability of each outcome once the second BhCG value was entered. Another common deviation was taking a second BhCG level despite having low progesterone at presentation. This was mostly done for clinical reasons, such as high initial BhCG or vaginal bleeding. Further, if the second blood sample was taken outside the target 48 ± 8 hour interval, centres were advised not to use M6, but second BhCG levels were recorded in the database nevertheless.

Biostatisticians (EC, BVC) and ultrasound examiners (SB, CK) performed data cleaning. Data cleaning included checking for outliers and inconsistencies, and sending queries to centres to retrieve missing information or to correct errors.

### Reference standard: PUL outcome

The reference standard for this validation study was the final outcome of the PUL as FPUL, IUP or EP (including PPUL). The final outcome was IUP when a gestation sac with an embryo was seen within the endometrial cavity. In cases with a negative urine pregnancy test at the 2‐week follow‐up phone consultation, the final outcome was FPUL. The final outcome was EP if a mass outside the endometrial cavity was confirmed on transvaginal sonography. The appearance of the EP was either an extra‐uterine gestation sac with a yolk sac (with or without visible embryo), an extra‐uterine gestation sac (‘bagel’ sign) or an inhomogeneous extra‐uterine mass (‘blob’ sign).^15^, ^16^ On the rare occasion that the pregnancy was never localised on transvaginal sonography, the PUL was classified as a PPUL when BhCG levels plateaued following at least three serum BhCG levels taken at approximately 48‐hour intervals (i.e. BhCG levels increased/decreased by <15% each time).

We are mainly interested in predicting EP. PPUL was considered as an EP event. Some patients were lost to follow up, such that the outcome was unknown. For the primary analysis, these patients were excluded.

### Prediction models and approaches evaluated in this study

We evaluated the 2ST (with M6P), M6P alone, M6NP alone, M4 and fixed BhCG ratio cut‐off values. We calculated model predictions post hoc based on the cleaned dataset, after imputation of missing values (see below). Model formulas for M6P, M6NP and M4 are given in Appendix S2. We did not evaluate the 2ST with M6NP because this only applies to patients taking progesterone supplements. For these patients, Step 1 can be used but the progesterone level is unreliable for use in M6P. This situation applied to a small number of patients in our sample (2%). For the 2ST, when the progesterone was ≤2 nmol/l, we used estimated risks of 0.961 for FPUL, 0.022 for IUP and 0.017 for EP. These risks were based on the full data of the original paper, which were also used to derive the final M6 coefficients.^12^ For 2ST, M6P, M6NP and M4, an estimated risk of EP ≥5% was used to classify patient at high risk of EP.

The use of BhCG ratio cut‐offs classifies patients without risk estimation. If the BhCG ratio was between 0.87 and 1.66, patients were classified as high risk of EP. If the BhCG ratio was <0.87, the PUL was classified as a FPUL. If the BhCG ratio was >1.66, the PUL was classified as an IUP.

### Sample size

There was no dedicated sample size determination for the study, given that it was a secondary analysis. The dataset contains over 300 PUL with a final outcome of EP. This was considered sufficient for this model validation study, based on common rules of thumb that at least 100–200 participants are needed in each outcome category.^17^, ^18^, ^19^

### Statistical analysis

Missing values were observed for initial progesterone, the second BhCG level and the final PUL outcome (for women who were lost to follow up). We assumed that missing values were ‘missing at random’, and used multiple imputation (100 imputations, see Appendix S3 for details). When the blood sample for the second BhCG measurement was not taken two calendar days after the initial blood sample, we assumed that the value was missing. When a patient was using progesterone supplements, we considered the progesterone value to be missing. Patients lost to follow up were included in the multiple imputation procedure to have their outcome imputed. However, these patients were excluded for the primary analysis.

For the assessment of discriminatory performance of 2ST, M6P, M6NP and M4, we calculated several areas under the receiver operating characteristic curve (AUC). AUCs quantify how well the estimated risks can discriminate between different outcomes. First, we calculated the binary AUC for EP versus FPUL/IUP based on the estimated risk of EP. Secondly, we calculated the AUC for FPUL versus IUP based on the conditional risk method.^20^ Thirdly, we calculated the polytomous discrimination index (PDI), which is a multinomial AUC that quantifies discrimination between the three outcome levels simultaneously.^21^ For every measure, we used meta‐analysis techniques to combine centre‐specific results. For the AUC for EP, we calculated 95% prediction intervals to quantify heterogeneity between centres.

Next, we evaluated the reliability/accuracy of the estimated risk of EP (calibration). We used logistic regression on the logit of the estimated risk of EP, and added random intercept and slope terms to account for clustering by centre. The results are presented as overall and centre‐specific smooth calibration curves, calibration intercepts and calibration slopes.^18^ The calibration intercept assesses whether estimated risks on average are too high (intercept <0) or too low (intercept >0). The calibration slope assesses whether estimated risks are too extreme (slope <1) or too moderate (slope >1). ‘Too extreme’ means that low estimated risks are too low and high estimated risks too high. ‘Too moderate’ means the opposite.

For every approach (including BhCG ratio cut‐offs), we calculated the percentage of PUL classified as low risk of EP, the percentage of PUL classified as low risk of EP that turned out to be FPUL/IUP (negative predictive value, NPV), the percentage of PUL classified as high risk of EP that turned out to be EP (positive predictive value, PPV), the percentage of EP classified as high risk of EP (sensitivity), and the percentage of FPUL/IUP classified as high risk of EP (false‐positive rate, FPR).

Finally, we assessed clinical utility for clinical decision making. This goes beyond discrimination and calibration, which are statistical measures to investigate the quality of the risk estimates. We used the Net Benefit to quantify the utility for identifying PUL that need close monitoring due to an increased risk of EP.^22^ Net Benefit calculates the net proportion of true positives: the proportion of true positives (EP classified as high risk) corrected for the proportion of false positives (FPUL/IUP classified as high risk). The latter is weighed by the risk threshold because preference for a lower risk threshold implies lower perceived harm of a false positive. Although we assume that a 5% risk of EP is a reasonable threshold to classify PUL as high risk for EP, we acknowledge that others may prefer a different threshold. Therefore, we calculated Net Benefit for thresholds from 3 to 10% and constructed decision curves (plot of Net Benefit by risk threshold) using meta‐analysis techniques to account for clustering by centre.^23^ These curves are compared with the default strategies of assuming everyone at high risk (‘treat all’) and assuming everyone at low risk (‘treat none’). If a model has lower Net Benefit than a default strategy at a specific threshold, the model is harmful at that threshold.^22^

We performed three sensitivity analyses. The first sensitivity analysis included the PUL that were lost to follow up. In all previous work, these cases were excluded, but at the moment the prediction is made, it is unknown who will be lost to follow up. The second sensitivity analysis used the observed second BhCG levels when they were taken 1–3 days after the initial measurement, and used imputed values when the second BhCG was taken after an interval of more than 3 days. This approach was used to develop M6.^12^ Finally, the original study set‐up allowed an assessment of the performance of the 2ST in an ‘as treated’ manner. The 2ST as treated refers to the final classification obtained in real time, irrespective of whether the 2ST protocol was followed correctly or data entry errors were present.

Further details on the statistical analysis are given in Appendix S3.

## Results

Data on 3272 women were available. After the exclusion of six patients that met exclusion criteria and 367 with an initial BhCG ≤25 IU/l, 2899 PULs remained (Tables 1 and S1, Figure S2). The median age of the women was 32 years (range 14–50). The median progesterone at presentation was 11 nmol/l (range 0.3–384), the median BhCG at presentation 519 IU/l (range 26–1534) and the median BhCG ratio was 0.90 (range 0.01–6.20). In all, 297 of 2899 women with PUL (10%) were lost to follow up, and had an unknown final outcome (Tables 1 and S1). Progesterone at presentation was missing for 8% of women, and second BhCG and BhCG ratio for 29% of women (Table 1). A total of 512 women with PUL had no second BhCG and 342 had a second BhCG that was not taken after a 2‐day interval (Table S2).

**Table 1:** Descriptive statistics

Variable	Result	All PUL (*n* = 2899)	PUL in primary analysis (*n* = 2602)	PUL lost to follow up (*n* = 297)
Age (y)	Missing, *n* (%)	2 (<1%)	2 (<1%)	0 (0%)
	Median (IQR)	32 (27–36)	32 (27–36)	31 (24–35)
	Range	14–50	14–50	15–48
Initial progesterone (nmol/l)	Missing, *n* (%)	298 (10%)	265 (10%)	33 (11%)
	Median (IQR)	11 (4–39)	12 (4–40)	7 (4–23)
	Range	0.3–333	0.3–219	1–333
Initial BhCG (IU/l)	Missing, *n* (%)	0 (0%)	0 (0%)	0 (0%)
	Median (IQR)	519 (174–1534)	518 (176–1508)	523 (163–1691)
	Range	26–126000	26–105006	26–126000
48h BhCG (IU/l)*	Missing, *n* (%)	854 (29%)	726 (28%)	128 (43%)
	Median (IQR)	520 (143–1389)	536 (143–1424)	382 (136–1278)
	Range	3–109 658	3–109 658	19–7762
BhCG ratio*	Missing, *n* (%)	854 (29%)	726 (28%)	128 (43%)
	Median (IQR)	0.90 (0.35–1.91)	0.97 (0.36–1.96)	0.44 (0.27–0.96)
	Range	0.01–6.20	0.01–6.20	0.01–3.00
Vaginal bleeding, *n* (%)	Missing	7/2899 (<1%)	7/2602 (<1%)	0 (0%)
	No	735/2892 (25%)	690/2595 (27%)	45 (15%)
	Minimal	802/2892 (28%)	724/2595 (28%)	78 (26%)
	Moderate	626/2892 (22%)	558/2595 (22%)	68 (23%)
	Soaked	365/2892 (13%)	320/2595 (12%)	45 (15%)
	Clots	364/2892 (13%)	303/2595 (12%)	61 (21%)
History of EP, *n* (%)	Missing	114/2899 (4%)	109/2602 (4%)	5/297 (2%)
	Yes	181/2785 (6%)	174/2493 (7%)	7/292 (2%)
	No	2604/2785 (94%)	2319/2493 (93%)	285/292 (98%)
Type of PUL, *n* (%)	Missing	5/2899 (<1%)	4/2602 (<1%)	1 (<1%)
	True PUL	1406/2894 (49%)	1291/2598 (50%)	115/296 (39%)
	Probable miscarriage	957/2894 (33%)	820/2598 (32%)	137 (46%)
	Probable IUP	389/2894 (13%)	354/2598 (14%)	35 (12%)
	Probable EP	142/2894 (5%)	133/2598 (5%)	9 (3%)

* Second BhCG values not taken 2 days after the initial value were considered missing values.

Descriptive statistics by PUL outcome for the current dataset and the original development data of M6 and 2ST^12^ suggest that data in both datasets were broadly similar, including the distribution of the final outcome (Table S3).

In the set of 2602 PUL that were used for our primary analysis, 334 were EP (including 72 PPUL), 930 IUP and 1338 FPUL. Overall results are presented here, and a full account of centre‐specific results using forest plots is available in the Supporting Information.

### Primary analysis: without PUL that were lost to follow up (*n* = 2602)

**Figure 1:**
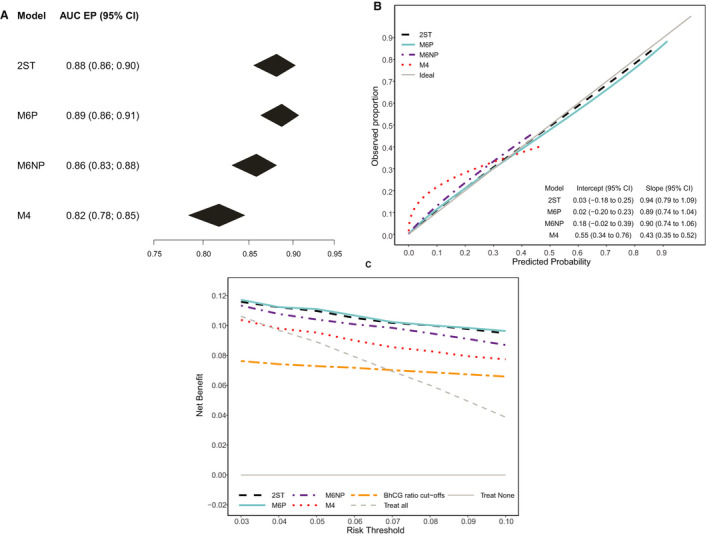
Key results of the primary analysis based on meta‐analysis of centre‐specific results (*n* = 2602). (A) Area under the receiver operating characteristic curve for ectopic pregnancy (EP) versus other. Higher values are better. (B) Calibration curves for the estimated risk of EP. Curves should ideally be on the diagonal line; when the curve is above the diagonal, estimated risks are too low and when the curve is below the diagonal, estimated risks are too high. (C) Decision curves based on the estimated risk of EP. Treat all and treat none refer to the default strategies of assuming everyone (treat all) or no one (treat none) is at high risk of EP. Higher Net Benefit is better; if Net Benefit does not exceed that of treat all and treat none, the model does not lead to better decisions than would a default strategy without any model.

**Figure 2:**
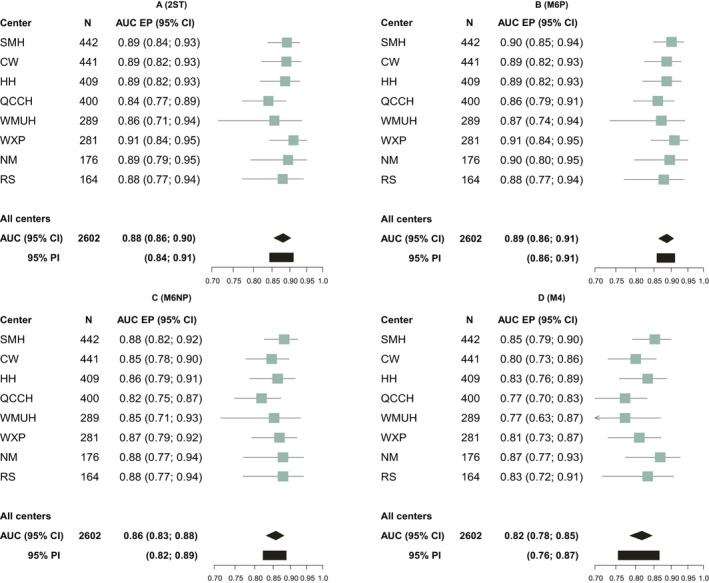
Forest plots of centre‐specific areas under the receiver operating characteristic curve (AUC) for ectopic pregnancy (EP) for 2ST (A), M6P (B), M6NP (C) and M4 (D). CI, confidence interval; PI, prediction interval; SMH, St Mary's; HH, Hillingdon; CW, Chelsea and Westminster; QCCH, Queen Charlotte's and Chelsea; WXP, Wexham Park; WMUH, West Middlesex University Hospital; NM, North Middlesex; RS, Royal Surrey.

The highest AUC for EP (versus other PUL) was obtained for M6P (AUC 0.89, 95% CI 0.86–0.91) (Figures 1 and 2). The AUC for EP was 0.88 (0.86–0.90) for 2ST, 0.86 (0.83–0.88) for M6NP, and 0.82 (0.78–0.85) for M4. Heterogeneity between centres was lowest for M6P (95% prediction interval 0.86–0.91) and largest for M4 (95% prediction interval 0.76–0.87) (Figure 2). The AUC for FPUL versus IUP was between 0.96 and 0.97 for all models (Figures S3 and S4). The PDI was highest for M6P (0.85) and 2ST (0.84), followed by M6NP (0.82) and M4 (0.80) (Figures S5 and S6).

Across centres, 2ST, M6P and M6NP had fairly well‐calibrated risk predictions for EP, as calibration curves were close to the diagonal line (Figure 1). Calibration slopes were between 0.89 and 0.94, and calibration intercepts between 0.02 and 0.18. M4 was poorly calibrated because risk estimates up to 0.35 were clearly too low (Figure 1). We observed limited heterogeneity in calibration between centres for every model (Figures S7–S11). The exception was West Middlesex, where we observed poorly calibrated risk predictions secondary to low EP prevalence.

**Figure 3:**
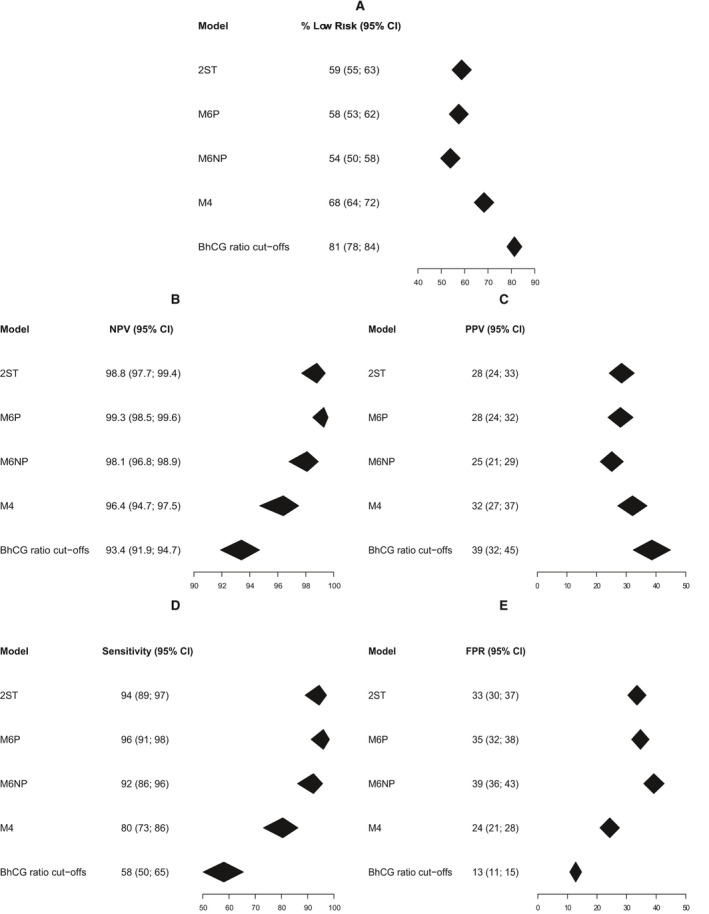
Classification results for the primary analysis (*n* = 2602). (A) Percentage of pregnancies of unknown location (PUL) classified as low risk. (B) Negative predictive value (percentage of failed PUL (FPUL) and intrauterine pregnancies (IUP) among PUL classified as low risk). (C) Positive predictive value (percentage of ectopic pregnancies [EP] among PUL classified as high risk). (D) Sensitivity for ectopic pregnancy (EP). (E) False‐positive rate (percentage of FPUL/IUP classified as high risk). False‐positive rate equals 100% minus specificity.

Step 1 of 2ST classified 16% (95% CI 12–20) of PUL as low risk (Figures 3 and S12). Importantly, one centre (*n* = 409) was excluded from this result because the assay kit was not sensitive enough. Overall, 81% PUL were classified as low risk by BhCG ratio cut‐offs, 68% by M4, 59% by 2ST and M6P, and 54% by M6NP. PPV varied between 25% (M6NP) and 39% (BhCG ratio cut‐offs) and NPV between 93% (BhCG ratio cut‐offs) and 99% (M6P and 2ST) (Figures S13 and S14). Sensitivity was highest for M6P (96%), followed by 2ST (94%), M6NP (92%), M4 (80%) and BhCG ratio cut‐offs (58%) (Figure S15). FPR was lowest for BhCG ratio cut‐offs (13%), followed by M4 (24%), 2ST (33%), M6P (35%) and M6NP (39%) (Figure S16).

The decision curve analysis (Figure 1) indicated that M6 and 2ST had the best clinical utility (highest Net Benefit) across the entire range of thresholds. M4 showed the poorest performance and was not better than assuming that everyone was at high risk of EP (‘treat all’) for risk thresholds ≤4%.

Box plots of risk predictions for each model are shown in Figures S17–S20.

### Sensitivity analyses

The sensitivity analysis that included the loss to follow‐up cases led to discrimination performances that were almost identical, albeit slightly lower (Figures S21–S24). The sensitivity analysis that used observed second BhCGs taken 1–3 days after the first also gave almost identical results, albeit slightly higher (Figures S25–S28).

Of the 2313 cases that were not lost to follow up and were not recruited at West Middlesex, there were 2147 (93%) that used 2ST (‘as treated’ group), of which 288 (13%) did not follow the 2ST protocol correctly (Figure S29). The classification performance of 2ST ‘as treated’ was again very similar as compared with the primary analysis (Figure S30). The main difference was that more PUL were classified as high risk, leading to higher sensitivity and false‐positive rates. This is likely reflected by the fact that Step 1 was sometimes not carried out (125 cases).

## Discussion

### Main findings

This multicentre validation study suggests that M6P is the best prediction model for PUL but that the combination of a single progesterone cut‐off with M6P (i.e. the 2ST) can make PUL management more efficient with little loss in performance. Furthermore, even without progesterone as a predictor, M6NP is a clear improvement over M4. The use of BhCG cut‐offs resulted in a low sensitivity for EP because this approach classified most PUL as low risk. The between‐centre heterogeneity in performance was modest. M6 is available at http://earlypregnancycare.co.uk/.

This is the first external validation study of M6 and the 2ST. In the original publication, the AUC to discriminate EP from other PUL was 0.90 for M6P and 0.87 for M6NP. For M4, the study reported an AUC of 0.85. Overall AUCs observed in the current study were slightly lower (0.89, 0.88 and 0.82, respectively). Calibration results were similar in both studies: M6P and M6NP showed good calibration, whereas M4 did not. In the current study, calibration for M4 was even worse.

### Strengths and limitations

The main strengths of our study are the large sample size, the assessment of between‐centre variability, and the evaluation of calibration and clinical utility. Further, we included PUL that were lost to follow up in a sensitivity analysis. The exclusion of lost to follow‐up cases is common but problematic because at the time the prediction is made, these patients are valid members of the target population. Although we included them using a thoughtful imputation procedure for the missing reference standard, the correctness of the assumptions underlying this procedure cannot be verified.

The key weakness of the study was that the data were collected to study the clinical implementation of the 2ST. This implied that a second BhCG measurement was not recommended when the initial progesterone was low, thereby increasing the amount of missing values. However, some cases with a low initial progesterone did have a second BhCG measurement. Discussions with local clinicians revealed that reasons for having a second BhCG were high initial BhCG, suspicion of EP, history of EP, presence of pain or bleeding, patient request or communication errors. It is therefore very likely that availability of a second BhCG can be explained by clinical information at presentation and random factors. Multiple imputation is suited for such ‘missingness mechanisms’,^24^ and allowed us to use data from all patients.

### Interpretation

The 2ST strategy classified 16% of PUL as low risk based on the progesterone at presentation. Hence, the 2ST would suggest that a second visit is not needed in about 1 in 6 cases of PUL, which makes follow up more efficient. The consequence is that the sensitivity for EP decreases slightly. The overall sensitivity observed in our study was 96% for M6P alone and 94% for the 2ST. The false‐positive rate, on the other hand, was slightly lower for the 2ST versus M6P (33% versus 35%). Seven of 334 EP (including PPUL) were classified as low risk at Step 1 in the 2ST. Of these seven, none required surgery (four managed expectantly, three received methotrexate). This suggests that the few EP that are missed due to very low progesterone at presentation are less problematic cases, although more data would be desirable.

Previously, a single progesterone cut‐off of ≤10 nmol/l has been suggested.^3^, ^4^ In our data, this would have classified 88/334 EP as low risk. One in three of these cases (28/88) underwent laparoscopy. We therefore believe that a cut‐off of 2 is clinically more acceptable, while still reducing follow up to one visit for 1 in 6 cases of PUL. We have to stress that one centre used a measurement kit for progesterone that was unable to detect very low levels of progesterone and had a lower limit of 4. It is therefore important to use a sufficiently sensitive measurement kit for progesterone. Furthermore, we used a risk threshold of 5% to select PUL as high risk of EP. Specifically, 5% implies that we allow up to 19 false positive per correctly identified EP.^22^ Other examiners may choose a different threshold if deemed more appropriate.

## Conclusion

M6P and its incorporation into a two‐step triage system based on a single progesterone cut‐off of ≤2 nmol/l perform well to predict PUL outcome and are clear improvements over M4 and simple BhCG cut‐offs. M6 and the 2ST are the recommended approaches for PUL triage.

### Disclosure of interests

BVC reports grants from Research Foundation – Flanders (FWO) and Internal Funds KU Leuven for the submitted work. TB reports grants, personal fees and non‐financial support from Samsung Medison, non‐financial support from Roche Diagnostics, non‐financial support from Abbott Diagnostics, all outside the submitted work. The remaining authors have no disclosures. Completed disclosure of interest forms are available to view online as Supporting Information.

### Contribution to authorship

EC, SB, CK, TB and BVC participated in the conception and design of the study. SB, JF, NMJ, CK, MAM, FA, BC, EK, OA, BG, SG, VV, CB, DG and CS acquired patient data. EC, LW and BVC wrote the statistical analysis plan and performed the statistical analysis. EC, SB, CK, DT, TB and BVC interpreted the results. EC, SB, CK, TB and BVC wrote the initial version of the manuscript. All authors critically revised the manuscript and approved the final version.

### Details of ethics approval

Consultation with a Research Ethics Committee and the research and development departments within each participating centre authorised the registration of the study as an audit, after guidance that formal ethics approval and written consent were not required from patients.

### Funding

EC, BVC and DT are supported by Research Foundation – Flanders (FWO) grant G0B4716N and Internal Funds KU Leuven grant C24/15/037. SB is supported by NIHR CLAHRC NWL (Collaboration for Leadership in Applied Health Research & Care, NorthWest London, grant RDIP033). TB is supported by the NIHR Biomedical Research Centre based at Imperial College Healthcare NHS Trust and Imperial College London. DT is Fundamental Clinical Researcher of FWO. The views expressed are those of the author(s) and not necessarily those of the NHS, the NIHR or the Department of Health. The funders had no role in study design, data collection, analysis and interpretation or reporting.

### Acknowledgements

None.

## Supporting information


**Figure S1.** Flowchart of the two‐step triage system (2ST).
**Figure S2.** Flowchart of patients.
**Figure S3.** Forest plots with centre‐specific areas under the receiver operating characteristic curve (AUC) for failed pregnancies of unknown location (FPUL) versus intrauterine pregnancies (IUP).
**Figure S4.** Summary forest plot of the area under the receiver operating characteristic curve (AUC) for failed pregnancies of unknown location (FPUL) versus intrauterine pregnancies (IUP). The diamonds refer to the meta‐analysis of centre‐specific results.
**Figure S5.** Forest plots of centre‐specific results for the Polytomous Discrimination Index (PDI).
**Figure S6.** Summary forest plot of the Polytomous Discrimination Index (PDI). The diamonds refer to the meta‐analysis of centre‐specific results.
**Figure S7.** Forest plots of centre‐specific calibration intercepts.
**Figure S8.** Summary forest plot of the calibration intercept. The diamonds refer to the meta‐analysis of centre‐specific results.
**Figure S9.** Forest plots of centre‐specific calibration slopes.
**Figure S10.** Summary forest plot of the calibration slope. The diamonds refer to the meta‐analysis of centre‐specific results.
**Figure S11.** Centre‐specific calibration curves.
**Figure S12.** Forest plots of centre‐specific percentages of patients classified as low risk.
**Figure S13.** Forest plots of centre‐specific negative predictive values (NPV).
**Figure S14.** Forest plots of centre‐specific positive predictive values (PPV).
**Figure S15.** Forest plots of centre‐specific sensitivities for ectopic pregnancy.
**Figure S16.** Forest plots of centre‐specific false positive rates (FPR).
**Figure S17.** Boxplots of estimated risks given by M6P.
**Figure S18.** Boxplots of estimated risks given by M6NP.
**Figure S19.** Boxplots of estimated risks given by 2ST.
**Figure S20.** Boxplots of estimated risks given by M4.
**Figure S21.** Summary forest plots of the area under the receiver operating characteristic curve (AUC) for ectopic pregnancy (EP), the AUC for failed pregnancies of unknown location (FPUL) versus intrauterine pregnancies (IUP), and the Polytomous Discrimination Index (PDI) based on all 2899 pregnancies of unknown location. The diamonds refer to the meta‐analysis of centre‐specific results.
**Figure S22.** Summary forest plots of the calibration intercept and calibration slope based on all 2899 pregnancies of unknown location. The diamonds refer to the meta‐analysis of centre‐specific results.
**Figure S23.** Summary calibration curves based on all 2899 pregnancies of unknown location.
**Figure S24.** Summary forest plots of the percentage of patients classified as low risk, the negative predictive value (NPV), the positive predictive value (PPV), the sensitivity for ectopic pregnancy, and the false positive rate (FPR) based on all 2899 pregnancies of unknown location. The diamonds refer to the meta‐analysis of centre‐specific results.
**Figure S25.** Summary forest plot of area under the receiver operating characteristic curve (AUC) for ectopic pregnancy (EP), the AUC for failed pregnancies of unknown location (FPUL) versus intrauterine pregnancies (IUP) and the Polytomous Discrimination Index (PDI) when using second beta human chorionic gonadotropin (BhCG) levels between 1 and 3 calendar days after the first. The diamonds refer to the meta‐analysis of centre‐specific results.
**Figure S26.** Summary forest plots of calibration intercept and calibration slope when using second beta human chorionic gonadotropin (BhCG) levels between 1 and 3 calendar days after the first. The diamonds refer to the meta‐analysis of centre‐specific results.
**Figure S27.** Summary calibration curves when using second beta human chorionic gonadotropin (BhCG) levels between 1 and 3 calendar days after the first.
**Figure S28.** Summary forest plots of the percentage of patients classified as low risk, the negative predictive value (NPV), the positive predictive value (PPV), the sensitivity for ectopic pregnancy and the false positive rate (FPR) when using second beta human chorionic gonadotropin (BhCG) levels between 1 and 3 calendar days after the first. The diamonds refer to the meta‐analysis of centre‐specific results.
**Figure S29.** Flowchart of the “as treated” analysis.
**Figure S30.** Centre‐specific forest plots of the percentage of patients classified as low risk, the negative predictive value (NPV), the positive predictive value (PPV), the sensitivity for ectopic pregnancy, and the false positive rate (FPR) of the “as treated” analysis of 2ST. The diamonds refer to the meta‐analysis of centre‐specific results.

**Table S1.** Descriptive statistics of key variables by centre.
**Table S2.** Interval between beta human chorionic gonadotropin (BhCG) measurements (in days).
**Table S3.** Descriptive statistics in original model development dataset of M6NP, M6P and 2ST and the current external validation dataset.

**Appendix S1.** Additional information on patient management based on 2ST.
**Appendix S2.** Detailed information about the prediction models.
**Appendix S3.** Imputation of missing data and statistical analysis.

Supplementary Material

Supplementary Material

Supplementary Material

Supplementary Material

Supplementary Material

Supplementary Material

Supplementary Material

Supplementary Material

Supplementary Material

Supplementary Material

Supplementary Material

Supplementary Material

Supplementary Material

Supplementary Material

Supplementary Material

Supplementary Material

Supplementary Material

Supplementary Material
