# Supplementary material for: External validation of models to predict the outcome of pregnancies of unknown location: a multicentre cohort study
Source: BJOG. 2020 Oct 7;128(3):552–62. doi: 10.1111/1471-0528.16497 (PMC7821217; doi:10.1111/1471-0528.16497)
Supplement: Supplementary file 1 — Figure S1. Flowchart of the two‐step triage system (2ST). Figure S2. Flowchart of patients. Figure S3. Forest plots with centre‐specific areas under the receiver operating characteristic curve (AUC) for failed pregnancies of unknown location (FPUL) versus intrauterine pregnancies (IUP). Figure S4. Summary forest plot of the area under the receiver operating characteristic curve (AUC) for failed pregnancies of unknown location (FPUL) versus intrauterine pregnancies (IUP). The diamonds refer to the meta‐analysis of centre‐specific results. Figure S5. Forest plots of centre‐specific results for the Polytomous Discrimination Index (PDI). Figure S6. Summary forest plot of the Polytomous Discrimination Index (PDI). The diamonds refer to the meta‐analysis of centre‐specific results. Figure S7. Forest plots of centre‐specific calibration intercepts. Figure S8. Summary forest plot of the calibration intercept. The diamonds refer to the meta‐analysis of centre‐specific results. Figure S9. Forest plots of centre‐specific calibration slopes. Figure S10. Summary forest plot of the calibration slope. The diamonds refer to the meta‐analysis of centre‐specific results. Figure S11. Centre‐specific calibration curves. Figure S12. Forest plots of centre‐specific percentages of patients classified as low risk. Figure S13. Forest plots of centre‐specific negative predictive values (NPV). Figure S14. Forest plots of centre‐specific positive predictive values (PPV). Figure S15. Forest plots of centre‐specific sensitivities for ectopic pregnancy. Figure S16. Forest plots of centre‐specific false positive rates (FPR). Figure S17. Boxplots of estimated risks given by M6P. Figure S18. Boxplots of estimated risks given by M6NP. Figure S19. Boxplots of estimated risks given by 2ST. Figure S20. Boxplots of estimated risks given by M4. Figure S21. Summary forest plots of the area under the receiver operating characteristic curve (AUC) for ectopic pregnancy (EP), the AUC for failed pregnancies of [file BJO-128-552-s001.pdf]

I. GENERAL STUDY INFORMATION

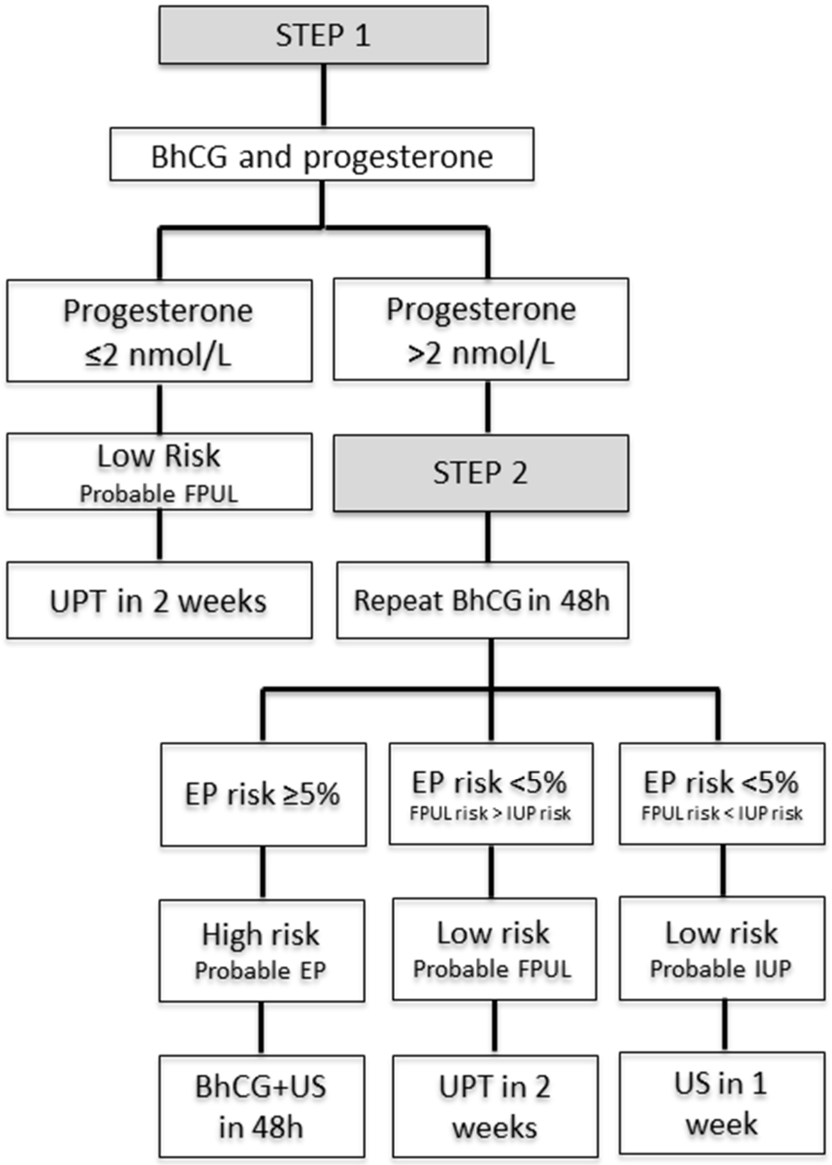

Figure S1. Flowchart of the two-step triage system (2ST).

BhCG, beta human chorionic gonadotropin; UPT, Urine Pregnancy Test; US, ultrasound; FPUL, failed pregnancy of unknown location; EP, ectopic pregnancy; IUP, intra-uterine pregnancy.

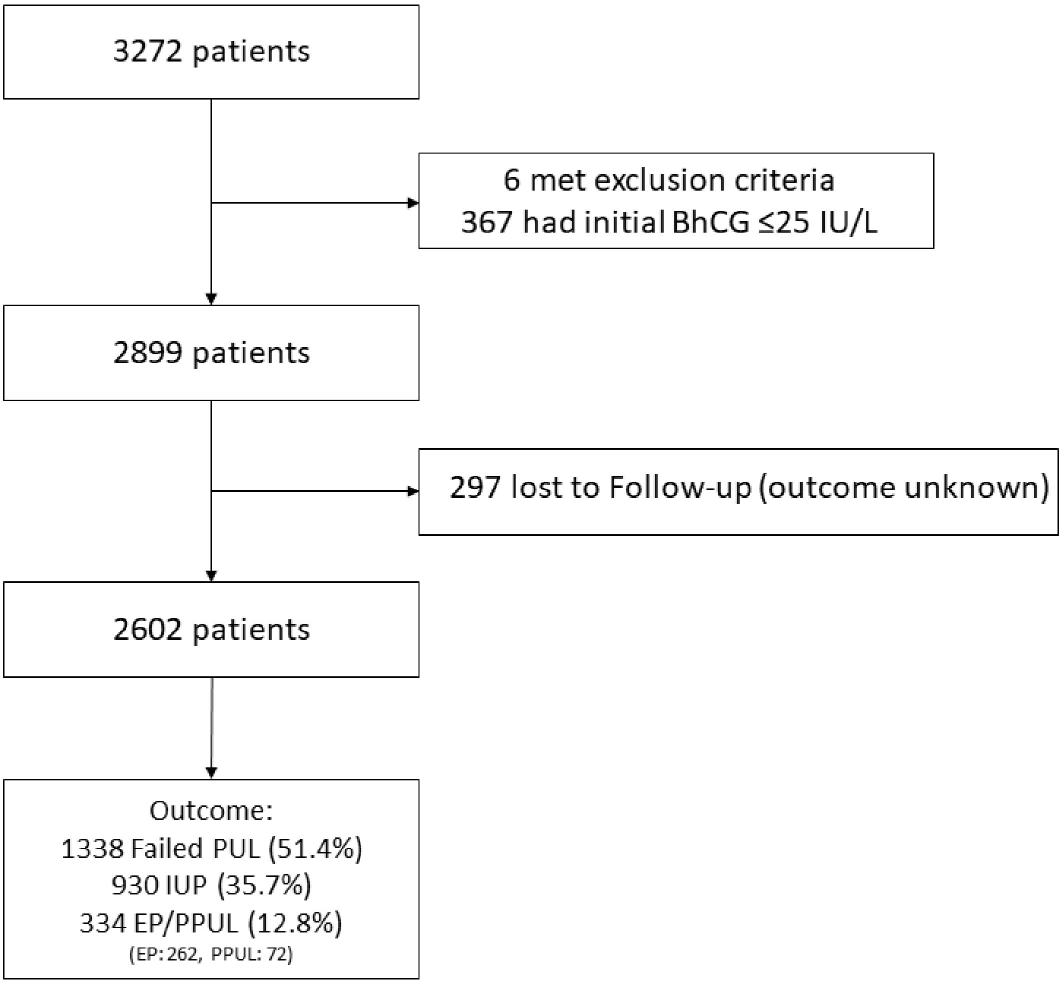

Figure S2. Flowchart of patients.

BhCG, beta human chorionic gonadotropin; FPUL, failed pregnancy of unknown location; EP, ectopic pregnancy; IUP, intra-uterine pregnancy; PPUL, Persisting pregnancy of unknown location

II. PRIMARY ANALYSIS (Lost to Follow up cases excluded)

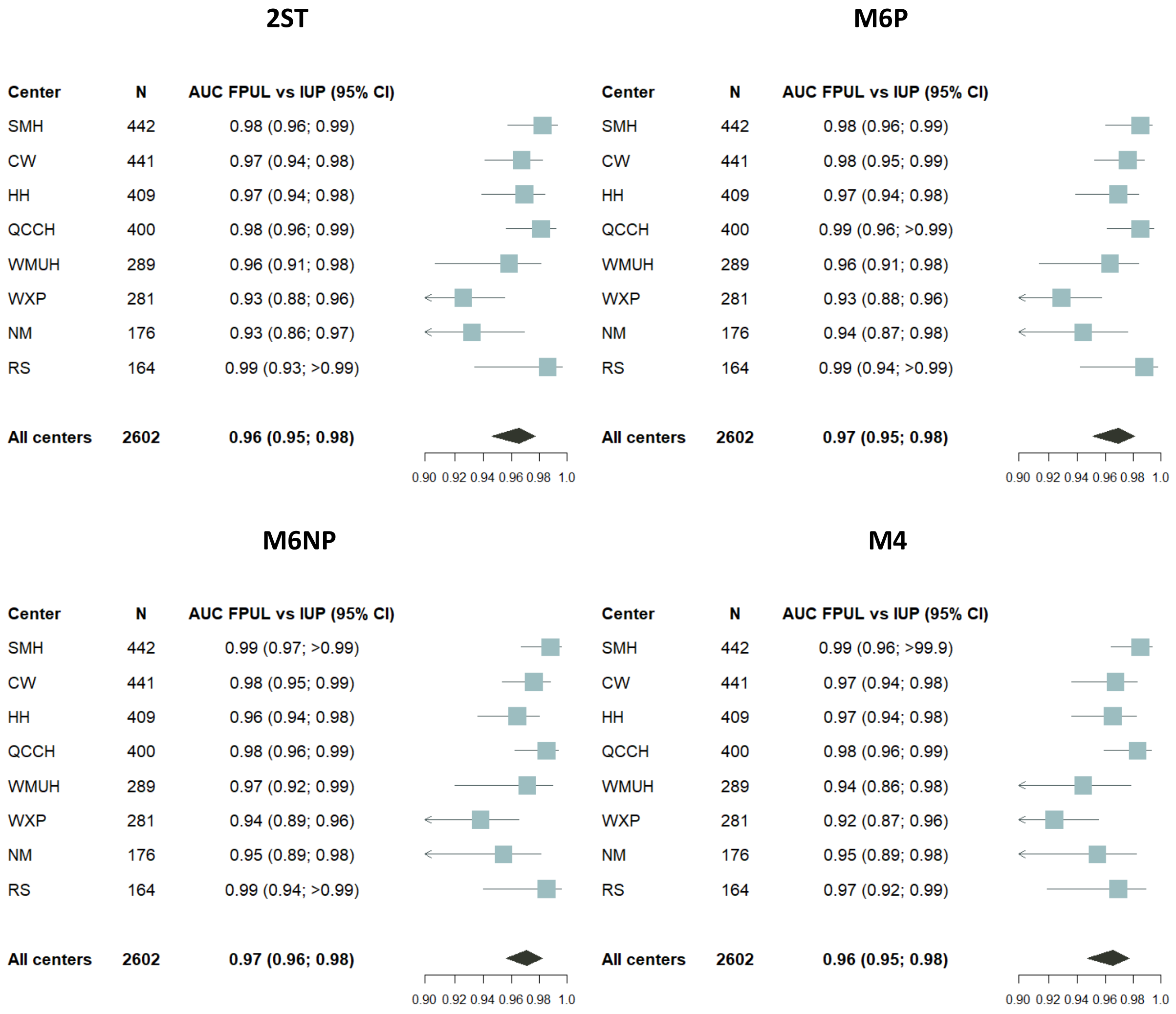

Figure S3. Forest plots with centre-specific areas under the receiver operating characteristic curve (AUC) for failed pregnancies of unknown location (FPUL) vs intra-uterine pregnancies (IUP).

CI, confidence interval; SMH, St. Mary's; HH, Hillingdon; CW, Chelsea and Westminster; QCCH, Queen Charlotte's and Chelsea; WXP, Wexham Park; WMUH, West Middlesex University Hospital; NM, North Middlesex; RS, Royal Surrey.

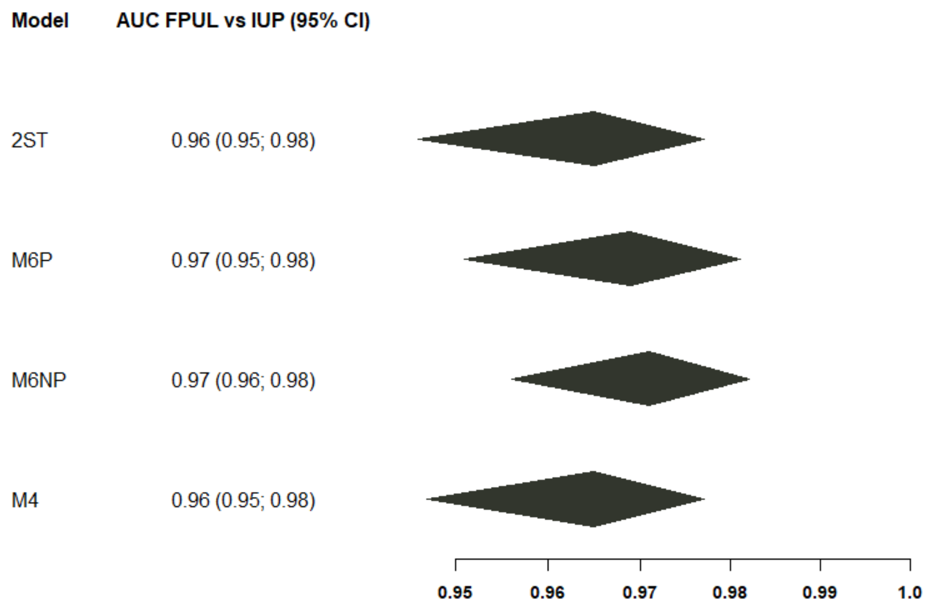

Figure S4. Summary forest plot of the area under the receiver operating characteristic curve (AUC) for failed pregnancies of unknown location (FPUL) vs intra-uterine pregnancies (IUP). The diamonds refer to the meta-analysis of centre-specific results.

CI, confidence interval.

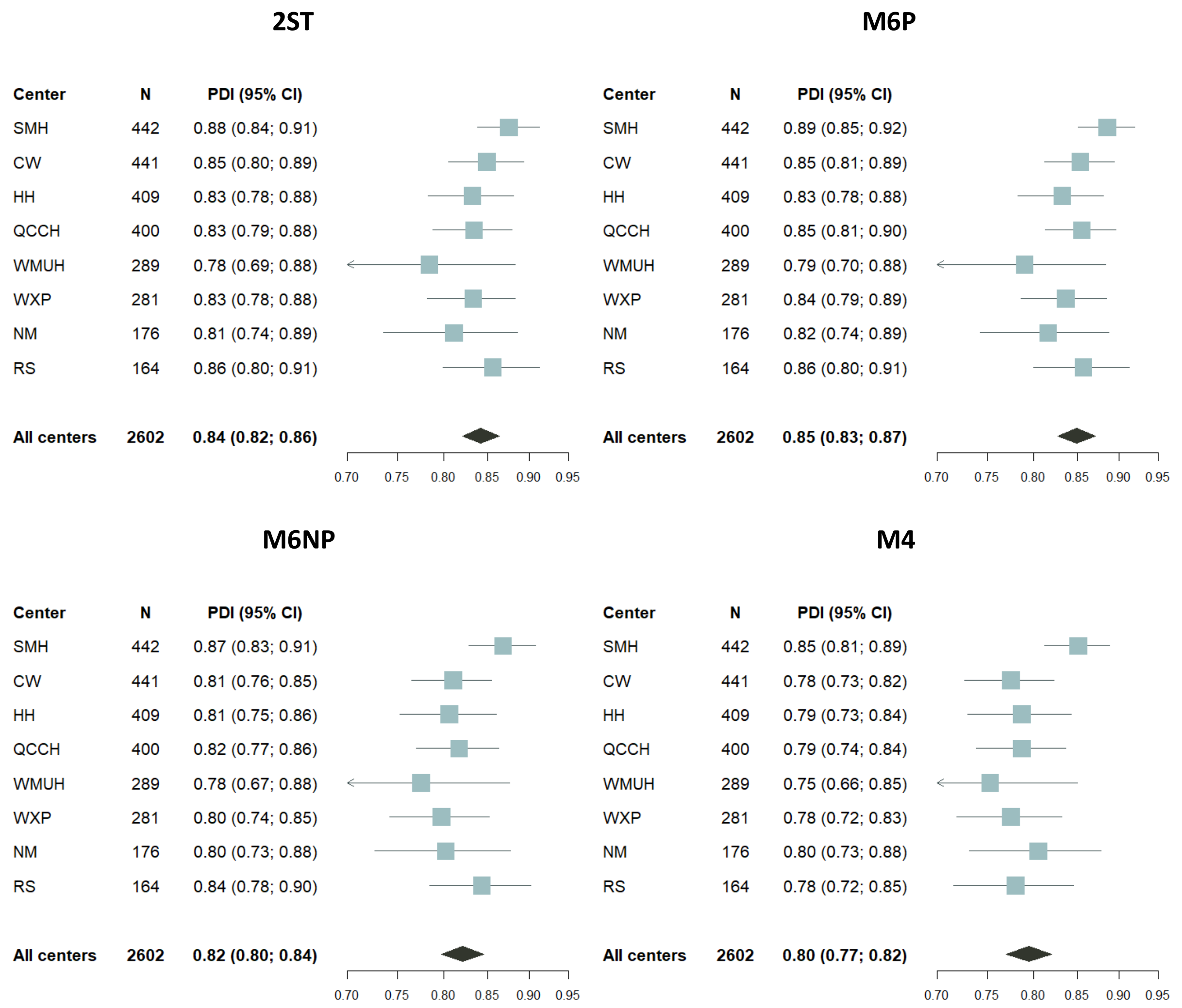

Figure S5. Forest plots of centre-specific results for the Polytomous Discrimination Index (PDI). CI, confidence interval; SMH, St. Mary's; HH, Hillingdon; CW, Chelsea and Westminster; QCCH, Queen Charlotte's and Chelsea; WXP, Wexham Park; WMUH, West Middlesex University Hospital; NM, North Middlesex; RS, Royal Surrey.

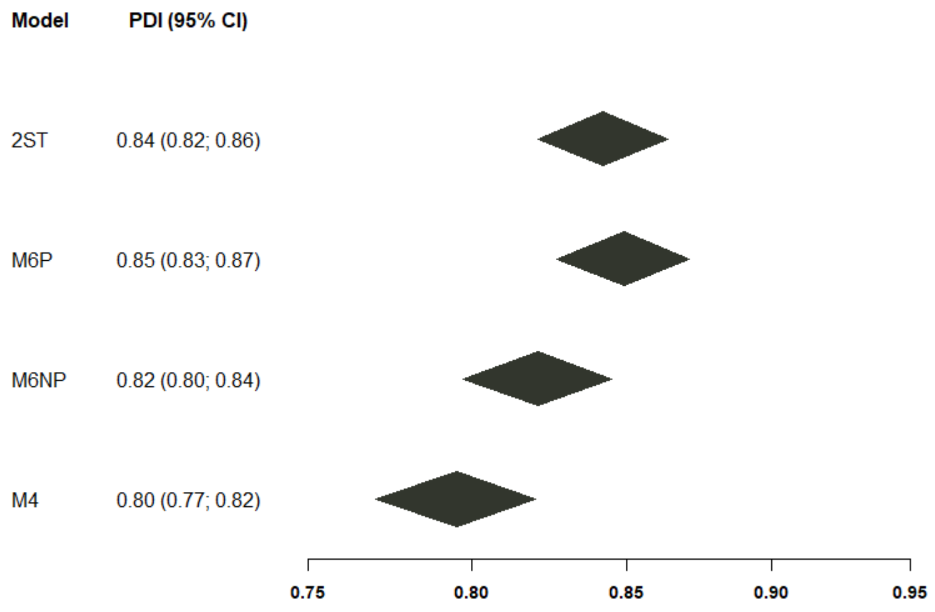

Figure S6. Summary forest plot of the Polytomous Discrimination Index (PDI). The diamonds refer to the meta-analysis of centre-specific results.

CI, confidence interval.

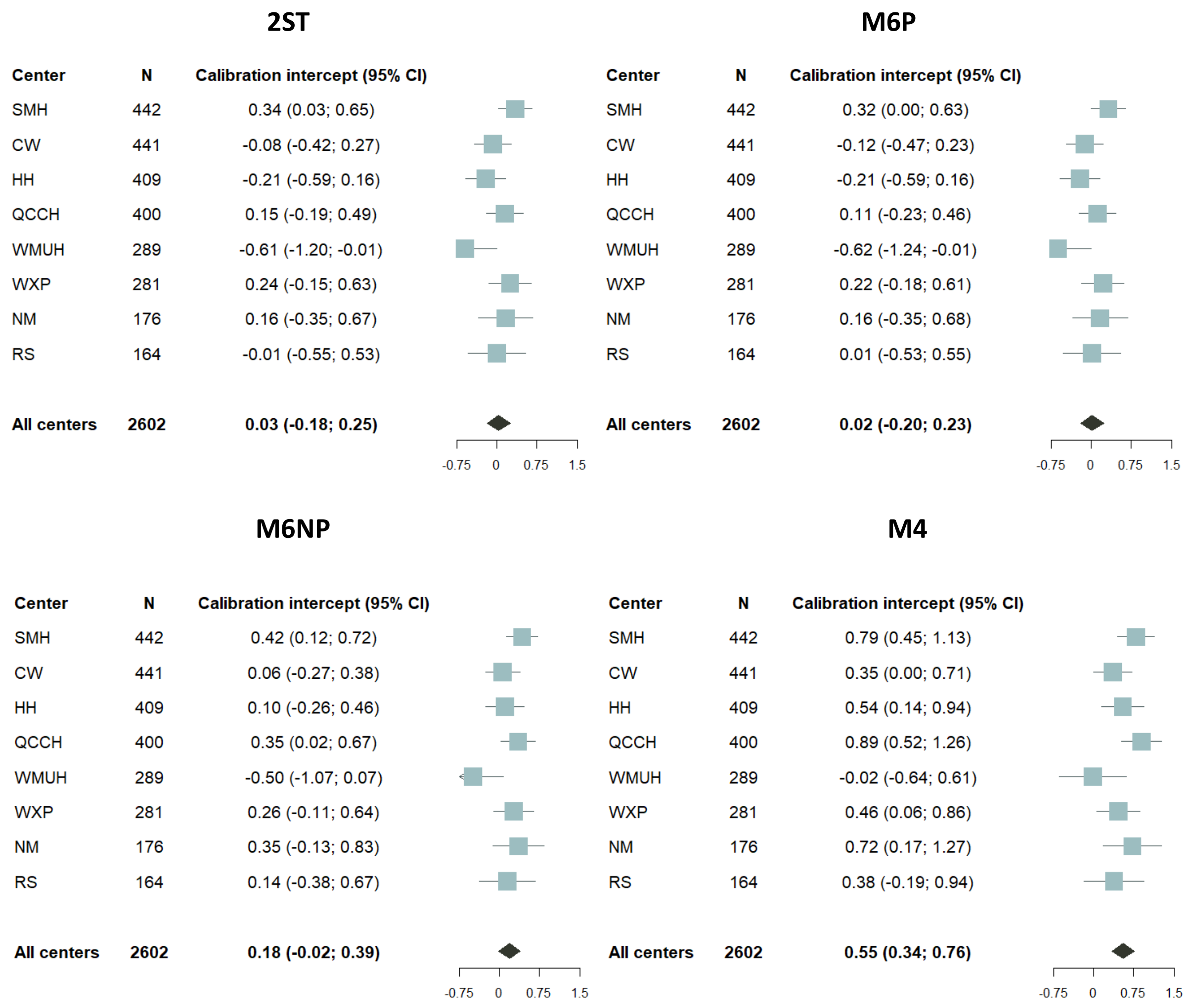

Figure S7. Forest plots of centre-specific calibration intercepts.

CI, confidence interval; SMH, St. Mary's; HH, Hillingdon; CW, Chelsea and Westminster; QCCH, Queen Charlotte's and Chelsea; WXP, Wexham Park; WMUH, West Middlesex University Hospital; NM, North Middlesex; RS, Royal Surrey.

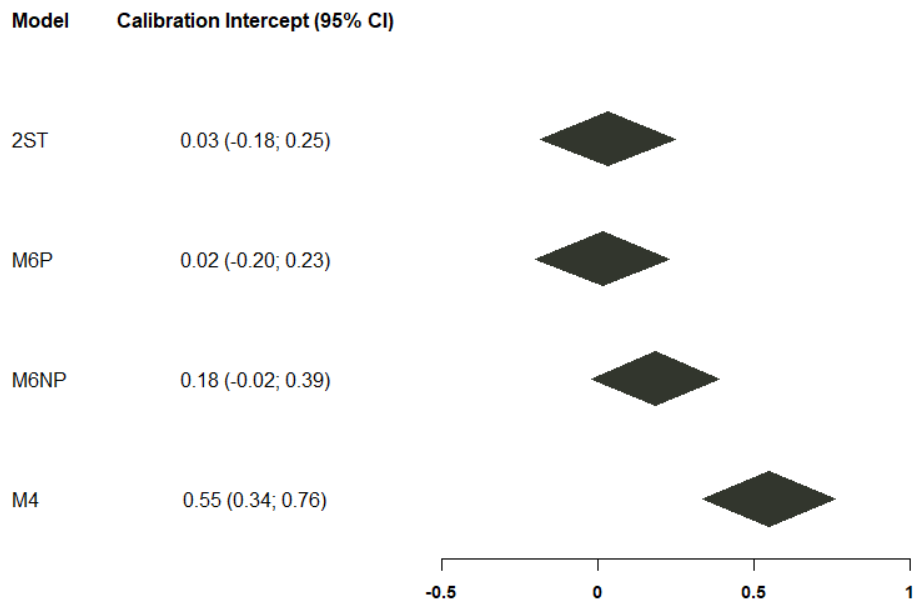

Figure S8. Summary forest plot of the calibration intercept. The diamonds refer to the meta-analysis of centre-specific results.

CI, confidence interval.

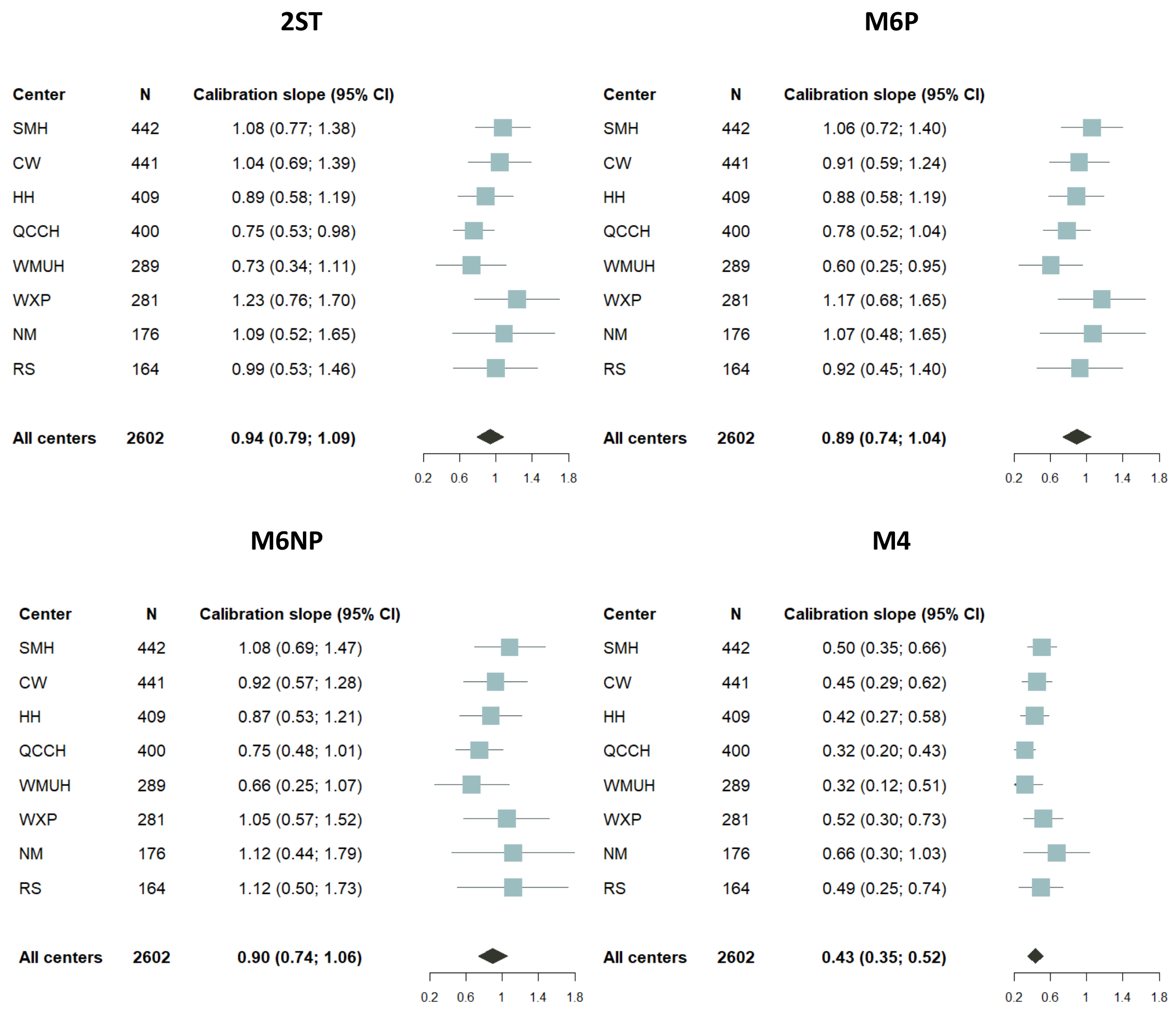

Figure S9. Forest plots of centre-specific calibration slopes.

CI, confidence interval; SMH, St. Mary's; HH, Hillingdon; CW, Chelsea and Westminster; QCCH, Queen Charlotte's and Chelsea; WXP, Wexham Park; WMUH, West Middlesex University Hospital; NM, North Middlesex; RS, Royal Surrey.

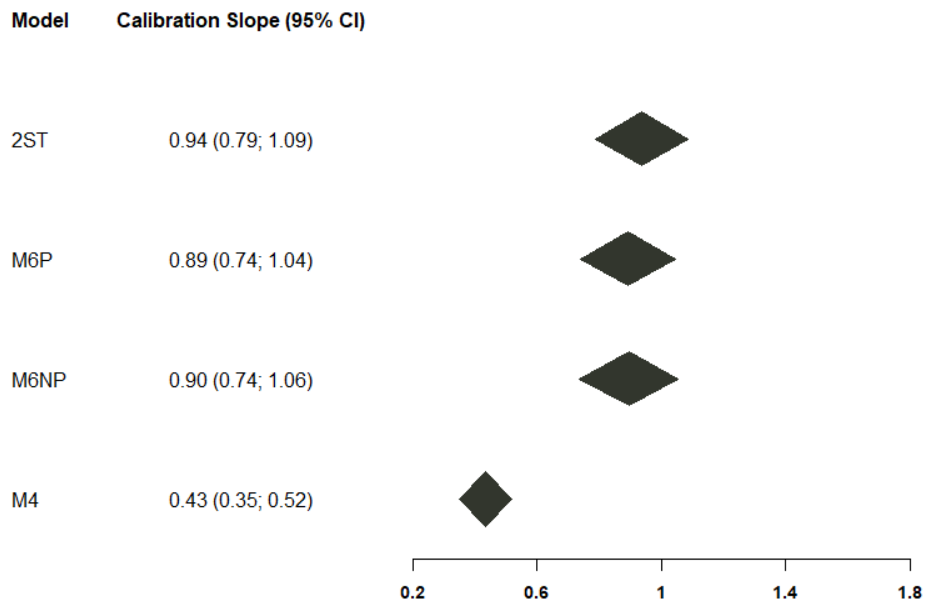

Figure S10. Summary forest plot of the calibration slope. The diamonds refer to the meta-analysis of centre-specific results.

CI, confidence interval.

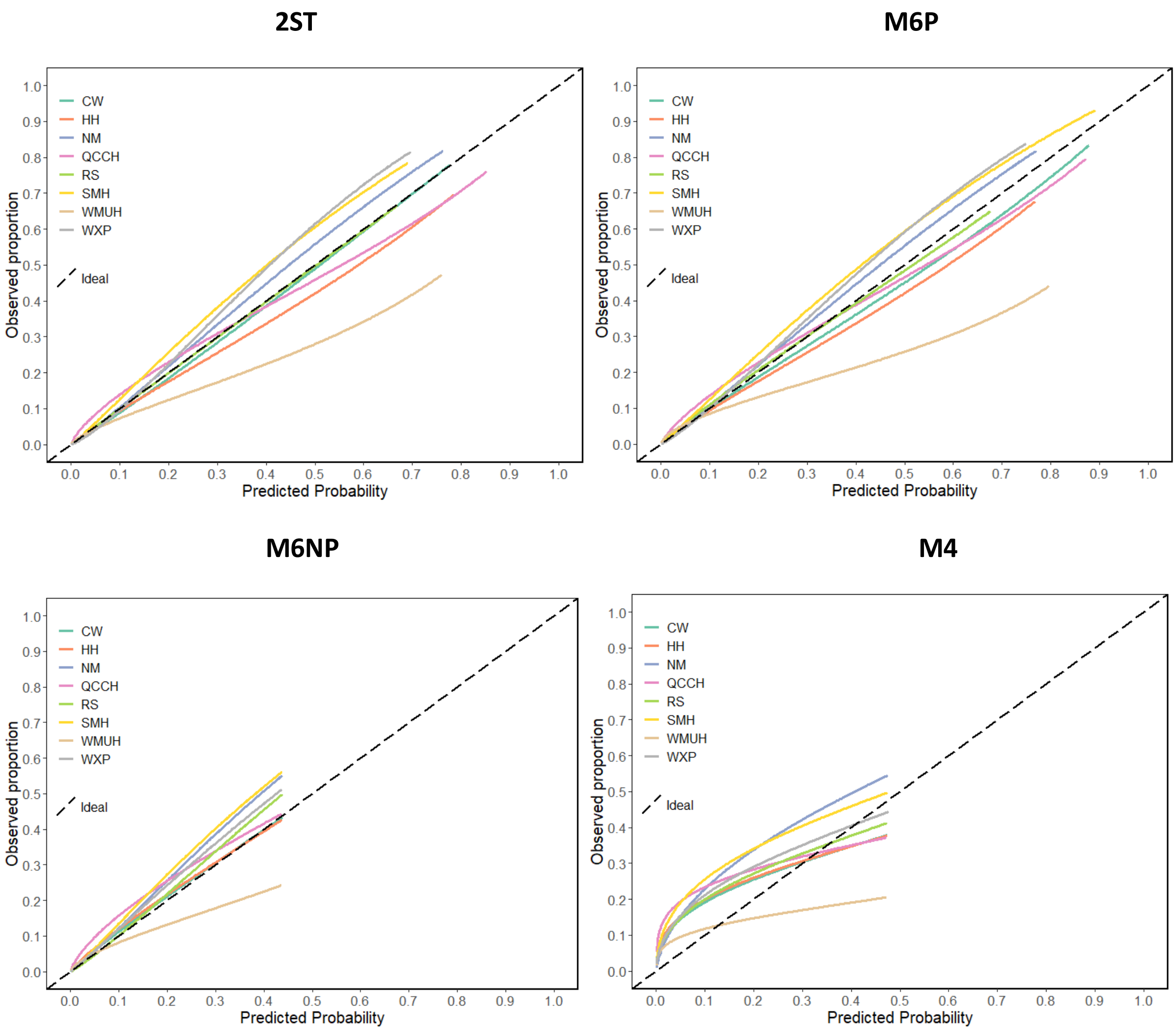

Figure S11. Centre-specific calibration curves.

SMH, St. Mary's; HH, Hillingdon; CW, Chelsea and Westminster; QCCH, Queen Charlotte's and Chelsea; WXP, Wexham Park; WMUH, West Middlesex University Hospital; NM, North Middlesex; RS, Royal Surrey.

2ST

Center	N	% Low Risk (95% CI)
SMH	442	55 % (50; 60)
CW	441	59 % (54; 64)
HH	409	59 % (54; 63)
QCCH	400	56 % (51; 61)
WMUH	289	73 % (67; 78)
WXP	281	55 % (49; 61)
NM	176	57 % (49; 65)
RS	164	57 % (49; 64)
**All centers**	**2602**	**59 % (55; 63)**

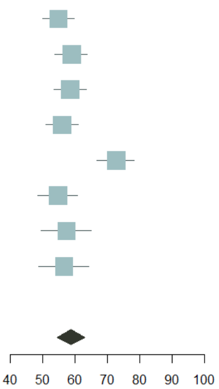

M6P

Center	N	% Low Risk (95% CI)
SMH	442	53 % (48; 58)
CW	441	57 % (52; 62)
HH	409	59 % (54; 63)
QCCH	400	54 % (49; 59)
WMUH	289	71 % (65; 77)
WXP	281	54 % (47; 60)
NM	176	56 % (48; 64)
RS	164	56 % (48; 64)
**All centers**	**2602**	**58 % (53; 62)**

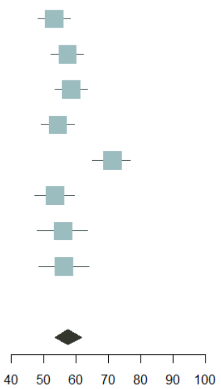

M6NP

Center	N	% Low Risk (95% CI)
SMH	442	50 % (45; 55)
CW	441	52 % (47; 57)
HH	409	58 % (53; 63)
QCCH	400	52 % (47; 57)
WMUH	289	67 % (59; 73)
WXP	281	47 % (41; 53)
NM	176	51 % (44; 59)
RS	164	56 % (48; 64)
**All centers**	**2602**	**54 % (50; 58)**

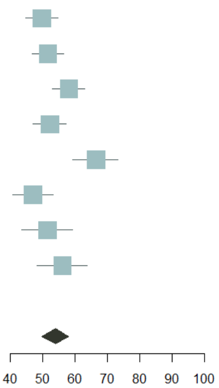

M4

Center	N	% Low Risk (95% CI)
SMH	442	66 % (61; 70)
CW	441	66 % (61; 70)
HH	409	73 % (68; 77)
QCCH	400	70 % (65; 74)
WMUH	289	79 % (73; 84)
WXP	281	59 % (53; 65)
NM	176	68 % (60; 75)
RS	164	66 % (59; 73)
**All centers**	**2602**	**68 % (64; 72)**

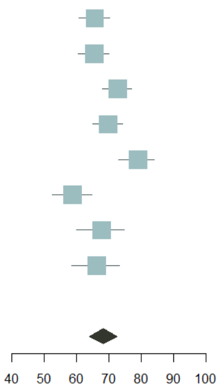

BhCG ratio: 0.87-1.66

Center	N	% Low Risk (95% CI)
SMH	442	79 % (75; 83)
CW	441	79 % (75; 83)
HH	409	84 % (80; 88)
QCCH	400	83 % (79; 86)
WMUH	289	90 % (85; 94)
WXP	281	77 % (71; 82)
NM	176	77 % (70; 83)
RS	164	79 % (72; 85)
**All centers**	**2602**	**81 % (78; 84)**

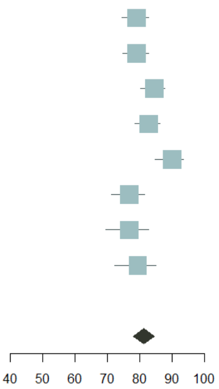

Figure S12. Forest plots of centre-specific percentages of patients classified as low risk.

BhCG, beta human chorionic gonadotropin; CI, confidence interval; SMH, St. Mary's; HH, Hillingdon; CW, Chelsea and Westminster; QCCH, Queen Charlotte's and Chelsea; WXP, Wexham Park; WMUH, West Middlesex University Hospital; NM, North Middlesex; RS, Royal Surrey.

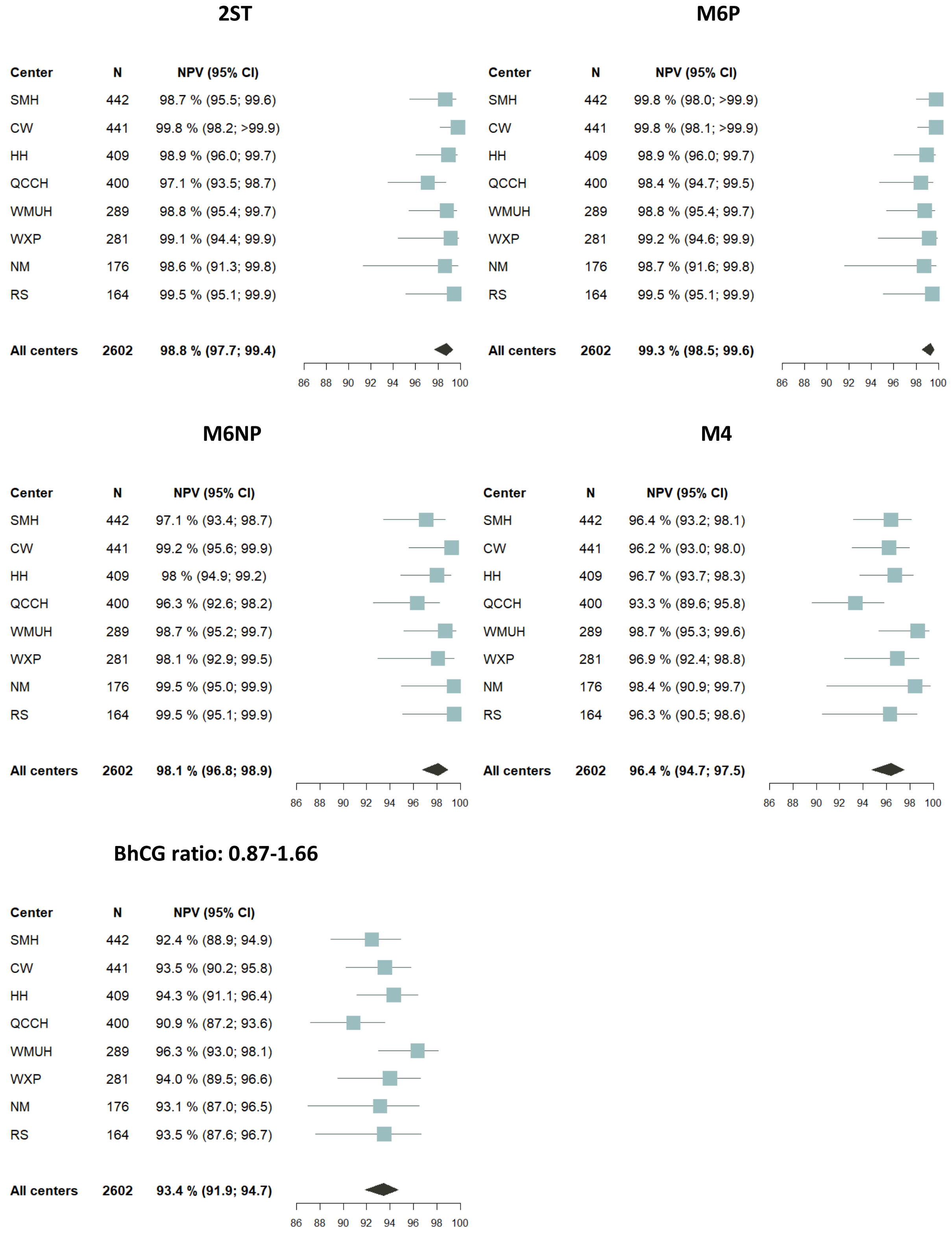

Figure S13. Forest plots of centre-specific negative predictive values (NPV).

BhCG, beta human chorionic gonadotropin; CI, confidence interval; SMH, St. Mary's; HH, Hillingdon; CW, Chelsea and Westminster; QCCH, Queen Charlotte's and Chelsea; WXP, Wexham Park; WMUH, West Middlesex University Hospital; NM, North Middlesex; RS, Royal Surrey.

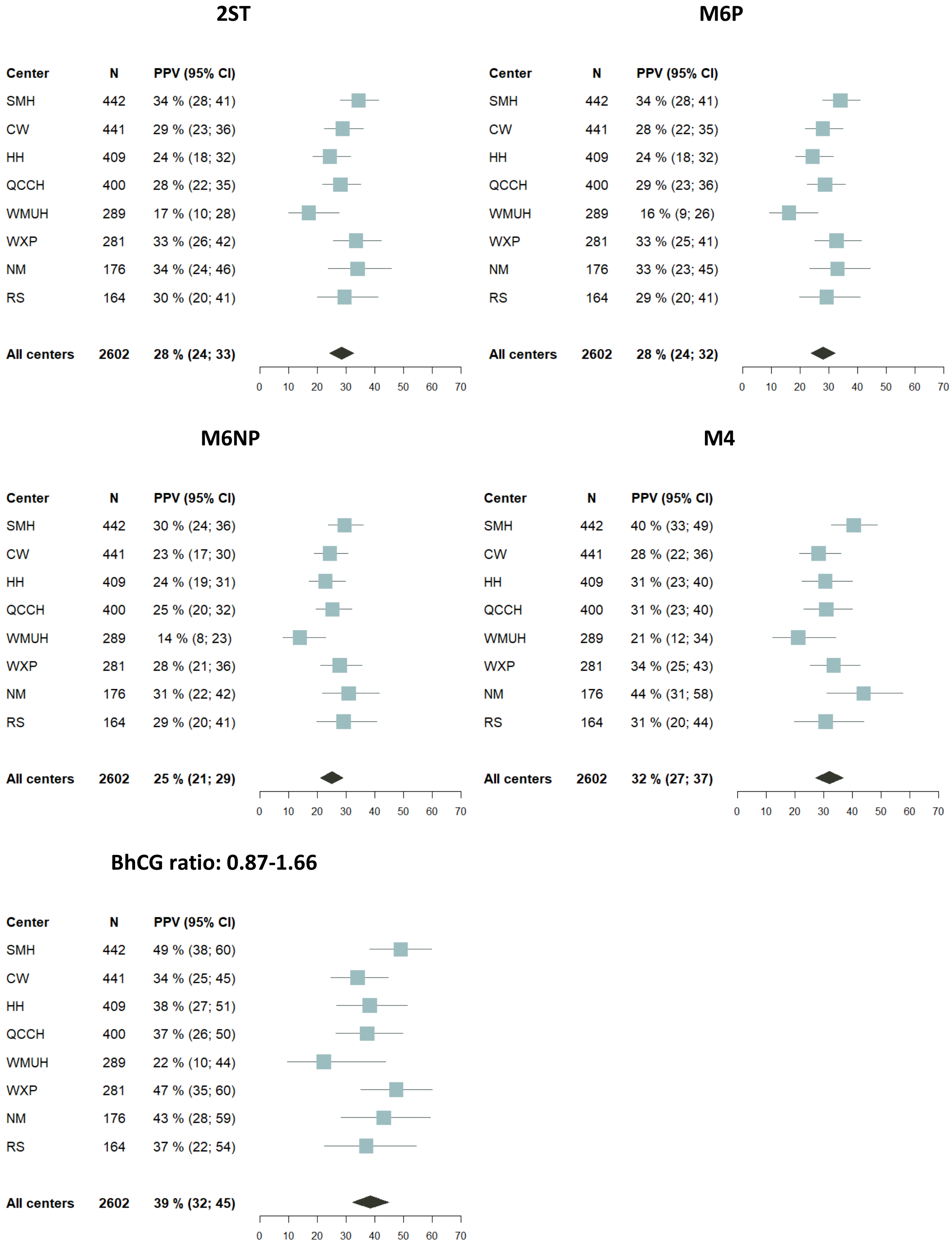

Figure S14. Forest plots of centre-specific positive predictive values (PPV).

BhCG, beta human chorionic gonadotropin; CI, confidence interval; SMH, St. Mary's; HH, Hillingdon; CW, Chelsea and Westminster; QCCH, Queen Charlotte's and Chelsea; WXP, Wexham Park; WMUH, West Middlesex University Hospital; NM, North Middlesex; RS, Royal Surrey.

2ST

Center	N	Sensitivity (95% CI)
SMH	442	95 % (85; 99)
CW	441	99 % (92; 99.9)
HH	409	94 % (80; 98)
QCCH	400	88 % (76; 95)
WMUH	289	84 % (54; 96)
WXP	281	97 % (82; 99.6)
NM	176	95 % (72; 99)
RS	164	98 % (82; 99.8)
**All centers**	**2602**	**94 % (89; 97)**

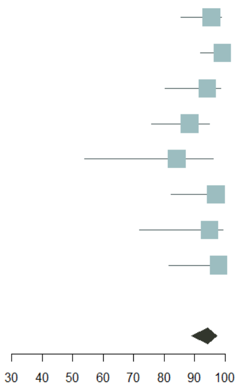

M6P

Center	N	Sensitivity (95% CI)
SMH	442	99 % (94; 99.9)
CW	441	99 % (92; 99.9)
HH	409	94 % (80; 98)
QCCH	400	94 % (81; 98)
WMUH	289	84 % (54; 96)
WXP	281	97 % (83; 99.6)
NM	176	95 % (73; 99)
RS	164	98 % (82; 99.8)
**All centers**	**2602**	**96 % (91; 98)**

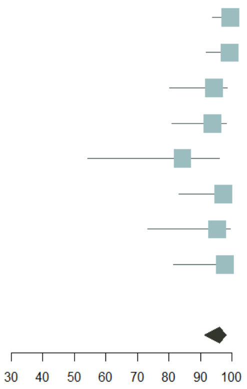

M6NP

Center	N	Sensitivity (95% CI)
SMH	442	91 % (81; 96)
CW	441	97 % (83; 99)
HH	409	89 % (75; 96)
QCCH	400	86 % (74; 93)
WMUH	289	84 % (55; 96)
WXP	281	94 % (80; 99)
NM	176	98 % (85; 99.8)
RS	164	98 % (82; 99.8)
**All centers**	**2602**	**92 % (86; 96)**

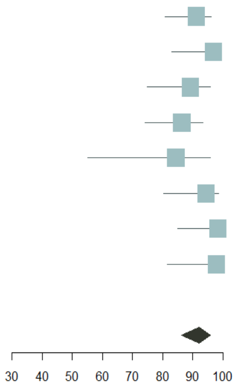

M4

Center	N	Sensitivity (95% CI)
SMH	442	85 % (74; 92)
CW	441	80 % (66; 89)
HH	409	78 % (62; 88)
QCCH	400	67 % (53; 78)
WMUH	289	81 % (50; 95)
WXP	281	88 % (74; 95)
NM	176	93 % (66; 99)
RS	164	81 % (59; 93)
**All centers**	**2602**	**80 % (73; 86)**

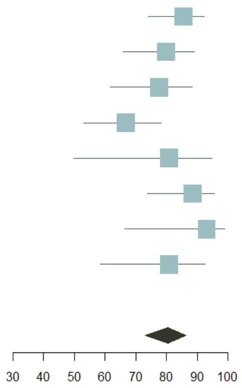

BhCG ratio: 0.87-1.66

Center	N	Sensitivity (95% CI)
SMH	442	63 % (50; 75)
CW	441	58 % (44; 71)
HH	409	55 % (39; 70)
QCCH	400	46 % (33; 59)
WMUH	289	40 % (17; 68)
WXP	281	70 % (54; 83)
NM	176	66 % (44; 82)
RS	164	59 % (38; 78)
**All centers**	**2602**	**58 % (50; 65)**

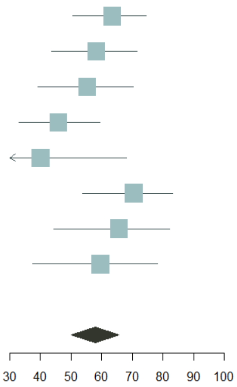

Figure S15. Forest plots of centre-specific sensitivities for ectopic pregnancy.

BhCG, beta human chorionic gonadotropin; CI, confidence interval; SMH, St. Mary's; HH, Hillingdon; CW, Chelsea and Westminster; QCCH, Queen Charlotte's and Chelsea; WXP, Wexham Park; WMUH, West Middlesex University Hospital; NM, North Middlesex; RS, Royal Surrey.

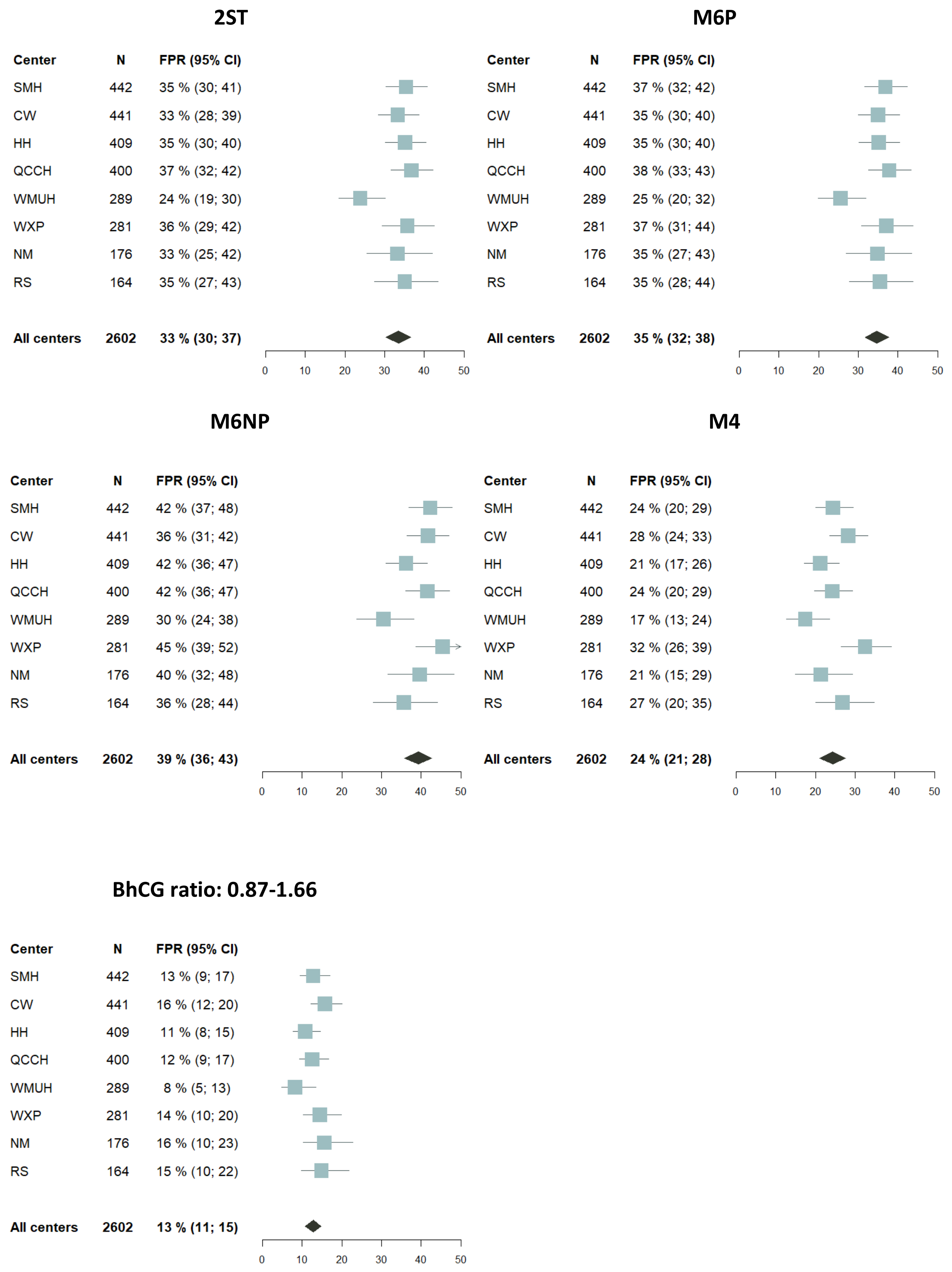

Figure S16. Forest plots of centre-specific false positive rates (FPR).

BhCG, beta human chorionic gonadotropin; CI, confidence interval; SMH, St. Mary's; HH, Hillingdon; CW, Chelsea and Westminster; QCCH, Queen Charlotte's and Chelsea; WXP, Wexham Park; WMUH, West Middlesex University Hospital; NM, North Middlesex; RS, Royal Surrey.

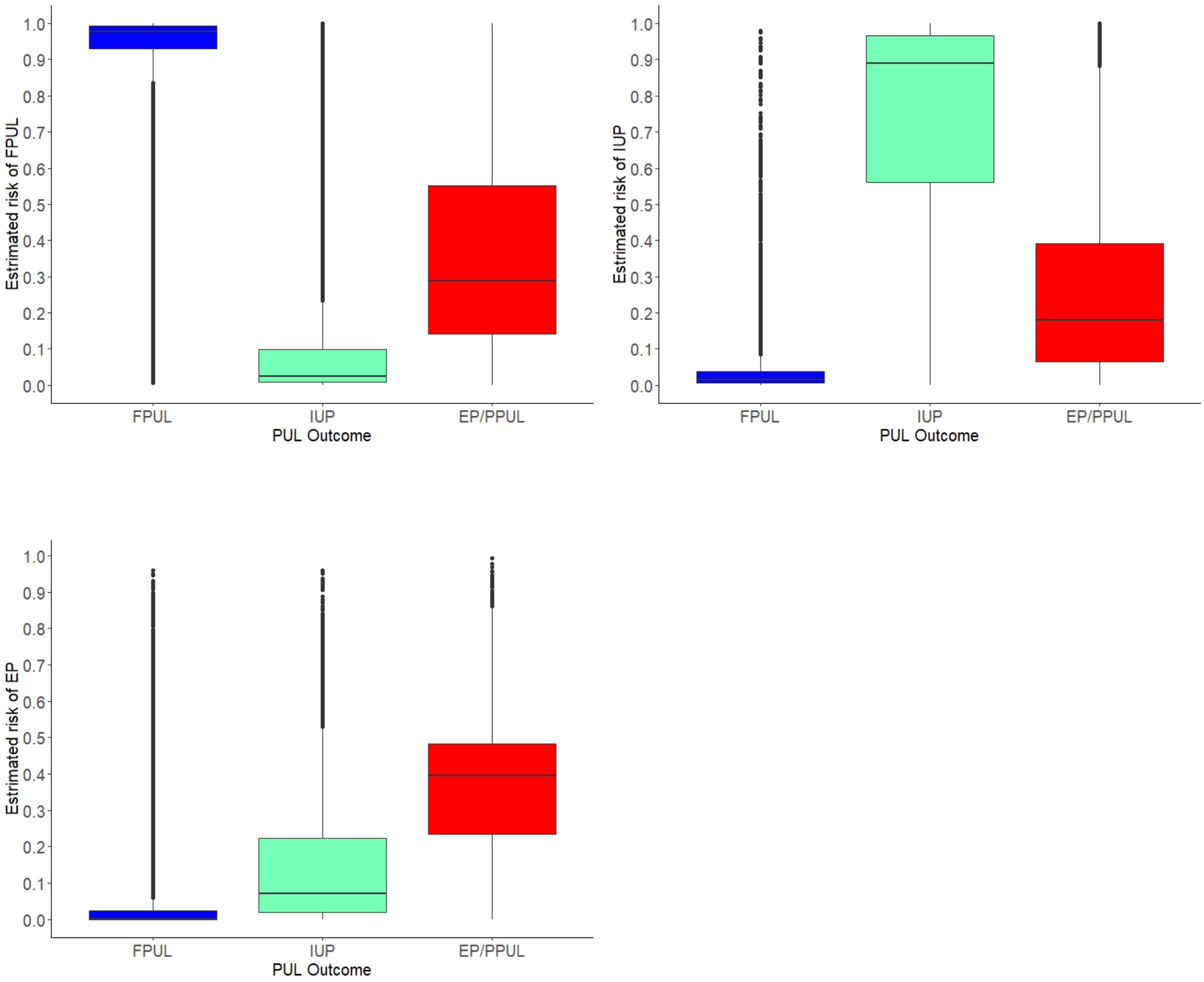

Figure S17. Boxplots of estimated risks given by M6P.

Results are based on a stacked dataset of the 100 completed datasets following multiple imputation.

FPUL, failed pregnancy of unknown location; EP, ectopic pregnancy; IUP, intra-uterine pregnancy.

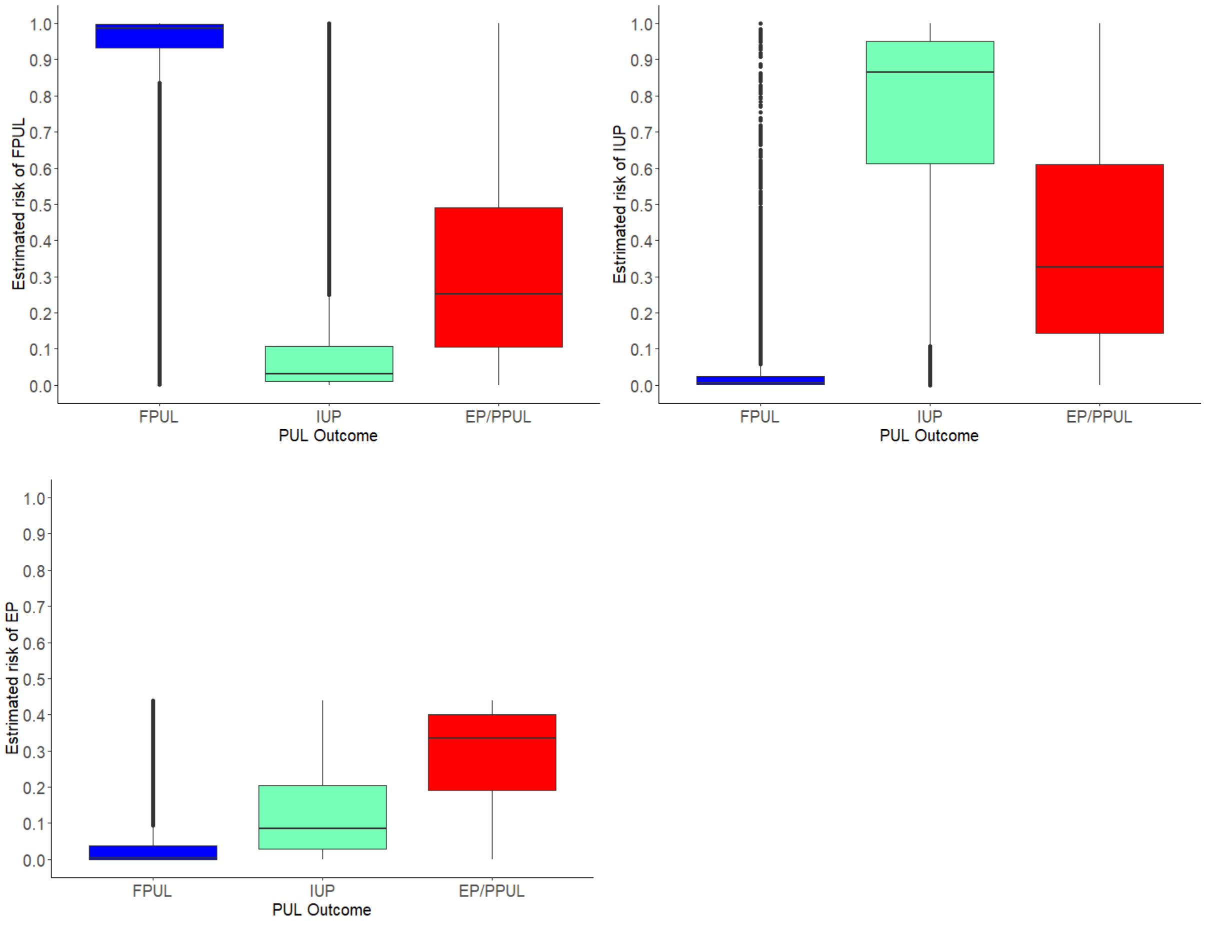

Figure S18. Boxplots of estimated risks given by M6NP.

Results are based on a stacked dataset of the 100 completed datasets following multiple imputation.

FPUL, failed pregnancy of unknown location; EP, ectopic pregnancy; IUP, intra-uterine pregnancy.

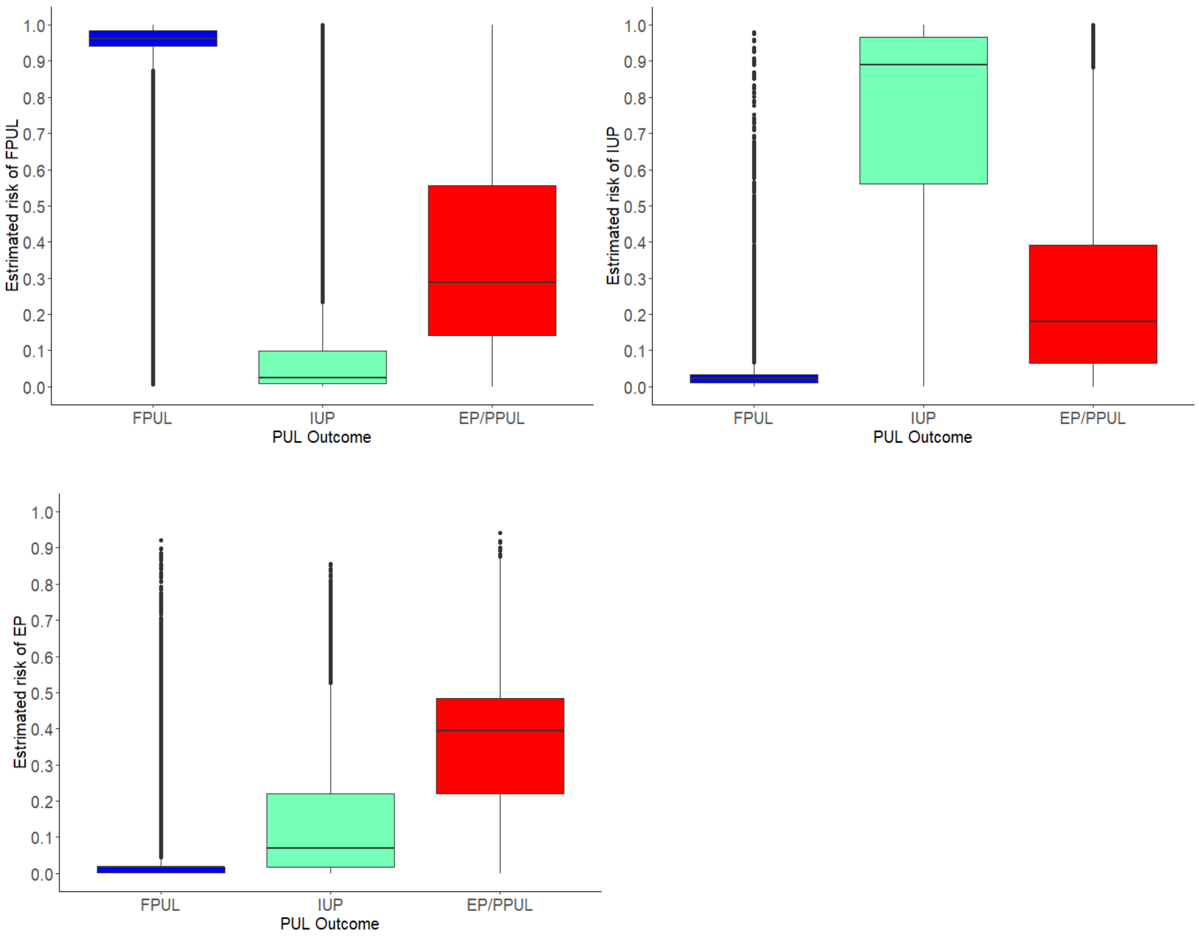

Figure S19. Boxplots of estimated risks given by 2ST.

Results are based on a stacked dataset of the 100 completed datasets following multiple imputation.

FPUL, failed pregnancy of unknown location; EP, ectopic pregnancy; IUP, intra-uterine pregnancy.

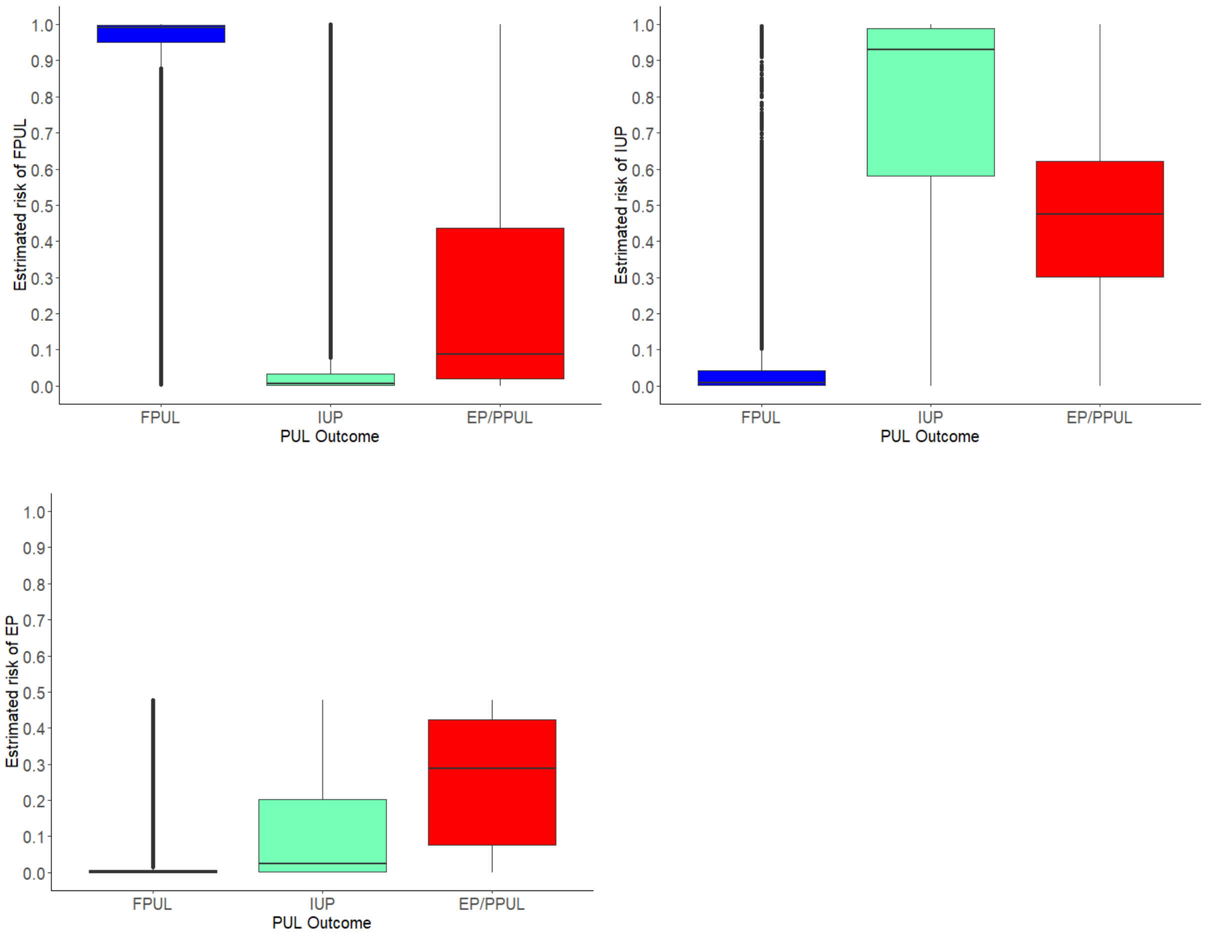

Figure S20. Boxplots of estimated risks given by M4.

Results are based on a stacked dataset of the 100 completed datasets following multiple imputation.

FPUL, failed pregnancy of unknown location; EP, ectopic pregnancy; IUP, intra-uterine pregnancy.

III. SENSITIVITY ANALYSES

i. Inclusion of pregnancies of unknown location that were lost to follow-up (n=2899)

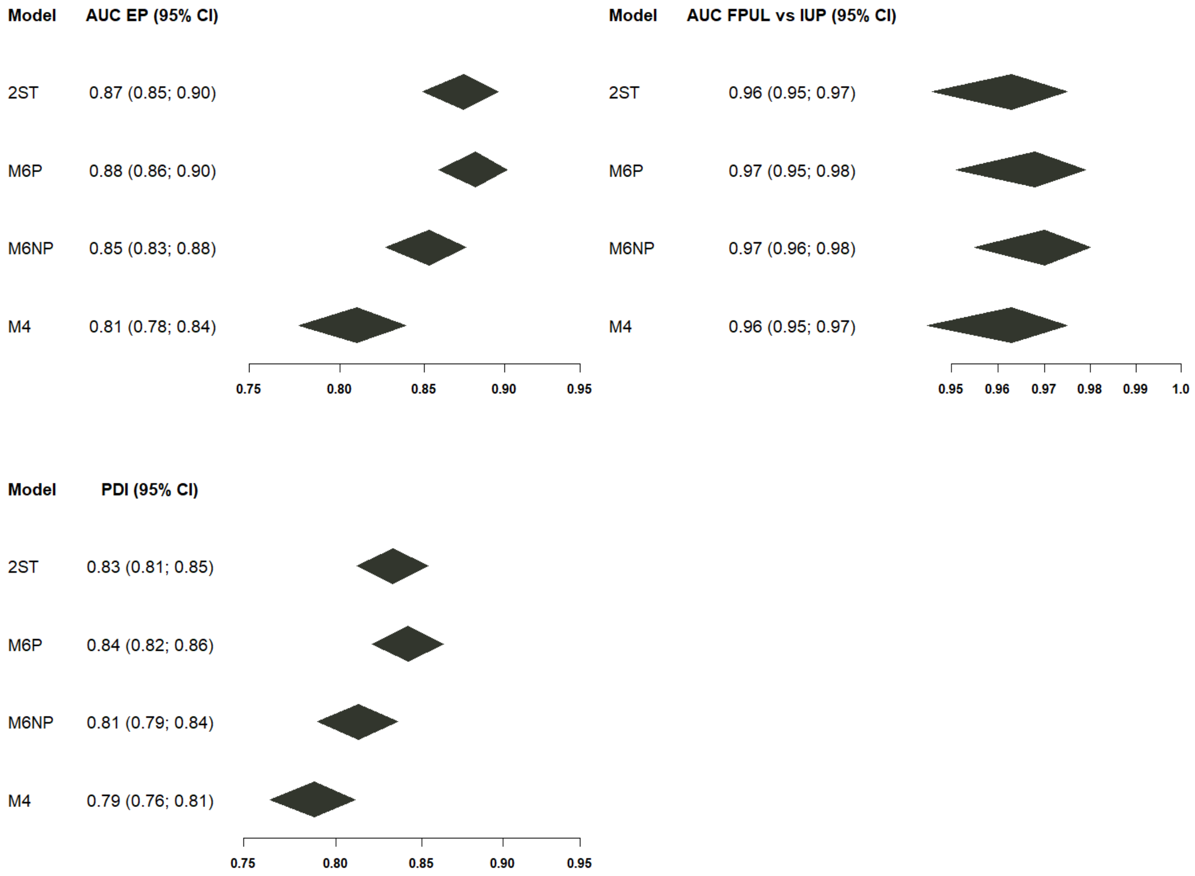

Figure S21. Summary forest plots of the area under the receiver operating characteristic curve (AUC) for ectopic pregnancy (EP), the AUC for failed pregnancies of unknown location (FPUL) vs intra-uterine pregnancies (IUP), and the Polytomous Discrimination Index (PDI) based on all 2899 pregnancies of unknown location. The diamonds refer to the meta-analysis of centre-specific results. CI, confidence interval.

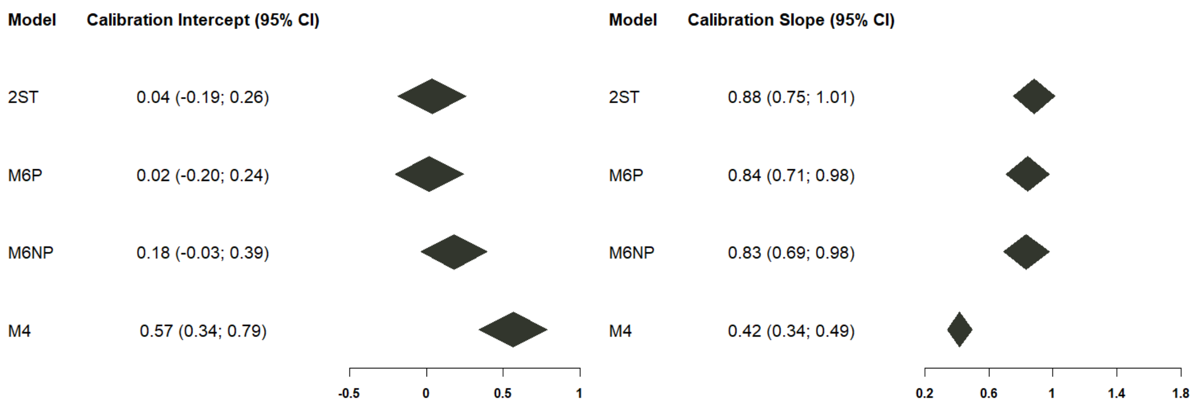

Figure S22. Summary forest plots of the calibration Intercept and calibration slope based on all 2899 pregnancies of unknown location. The diamonds refer to the meta-analysis of centre-specific results. CI, confidence interval.

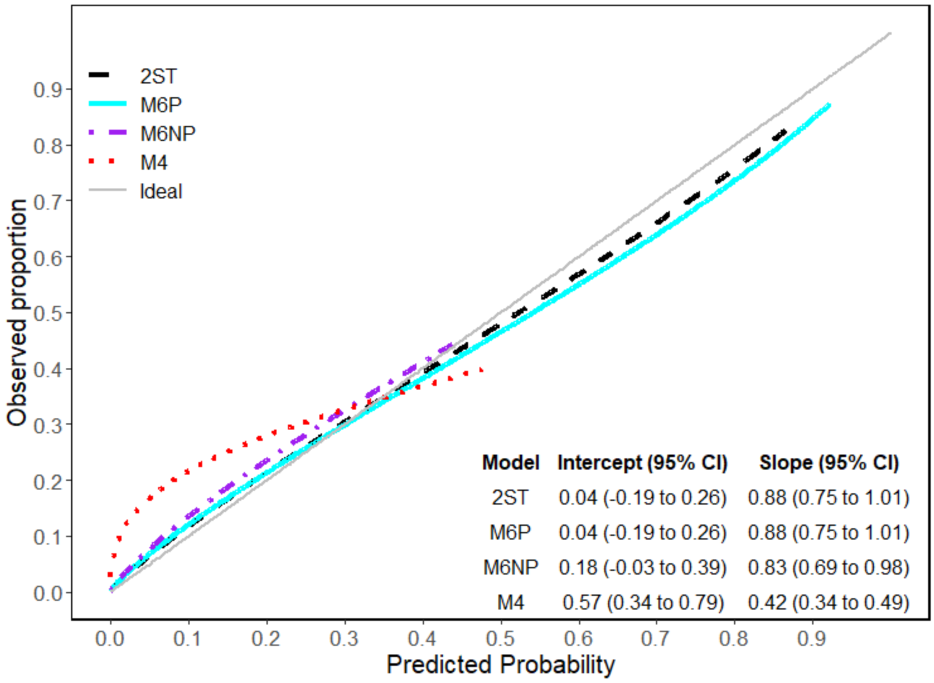

Figure S23. Summary calibration curves based on all 2899 pregnancies of unknown location. CI, confidence interval.

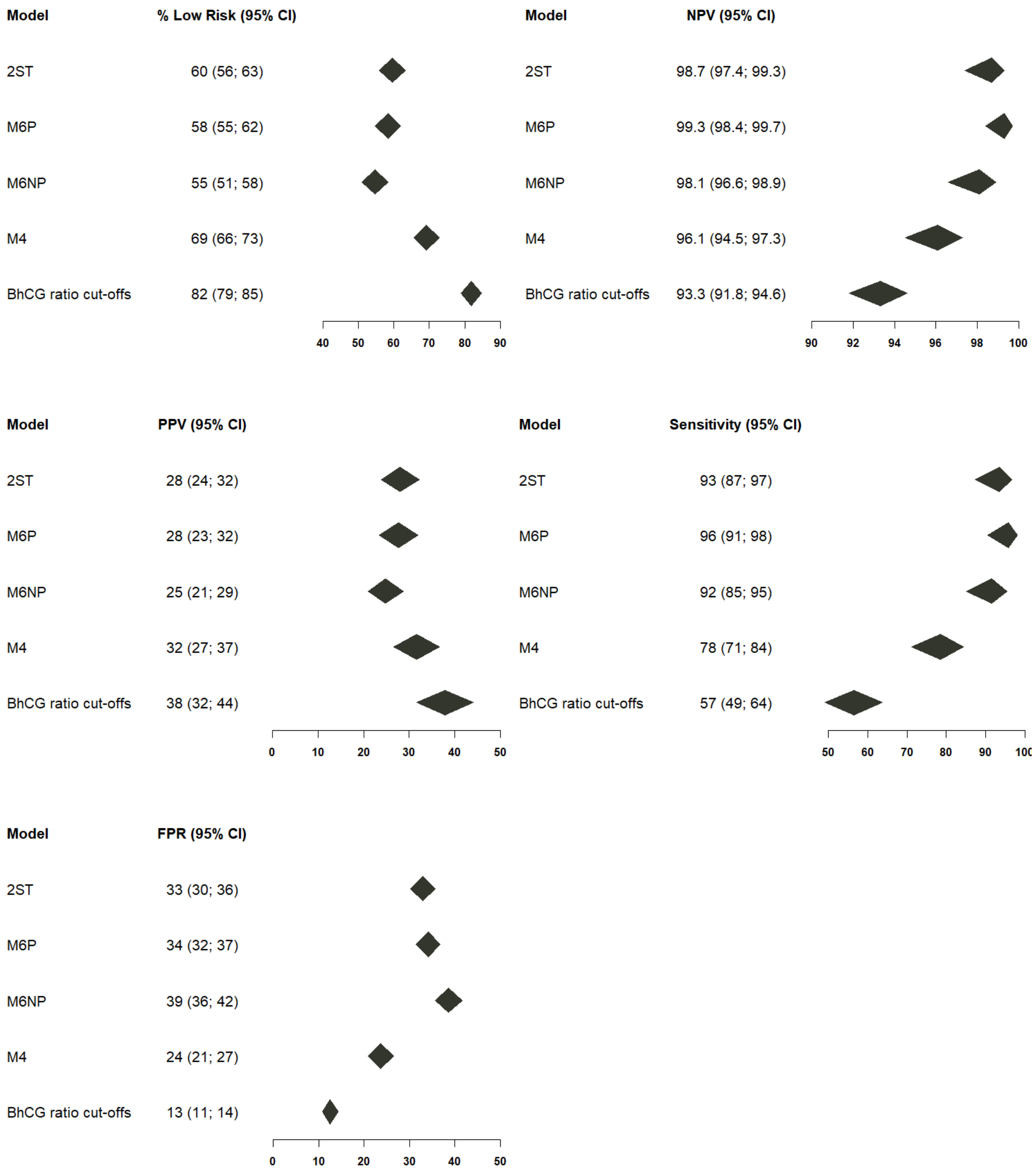

Figure S24. Summary forest plots of the percentage of patients classified as low risk, the negative predictive value (NPV), the positive predictive value (PPV), the sensitivity for ectopic pregnancy, and the false positive rate (FPR) based on all 2899 pregnancies of unknown location. The diamonds refer to the meta-analysis of centre-specific results.

BhCG, beta human chorionic gonadotropin; CI, confidence interval.

ii. Inclusion of second beta human chorionic gonadotropin (BhCG) measurements between 1 to 3 calendar days after the initial measurement (n=2602)

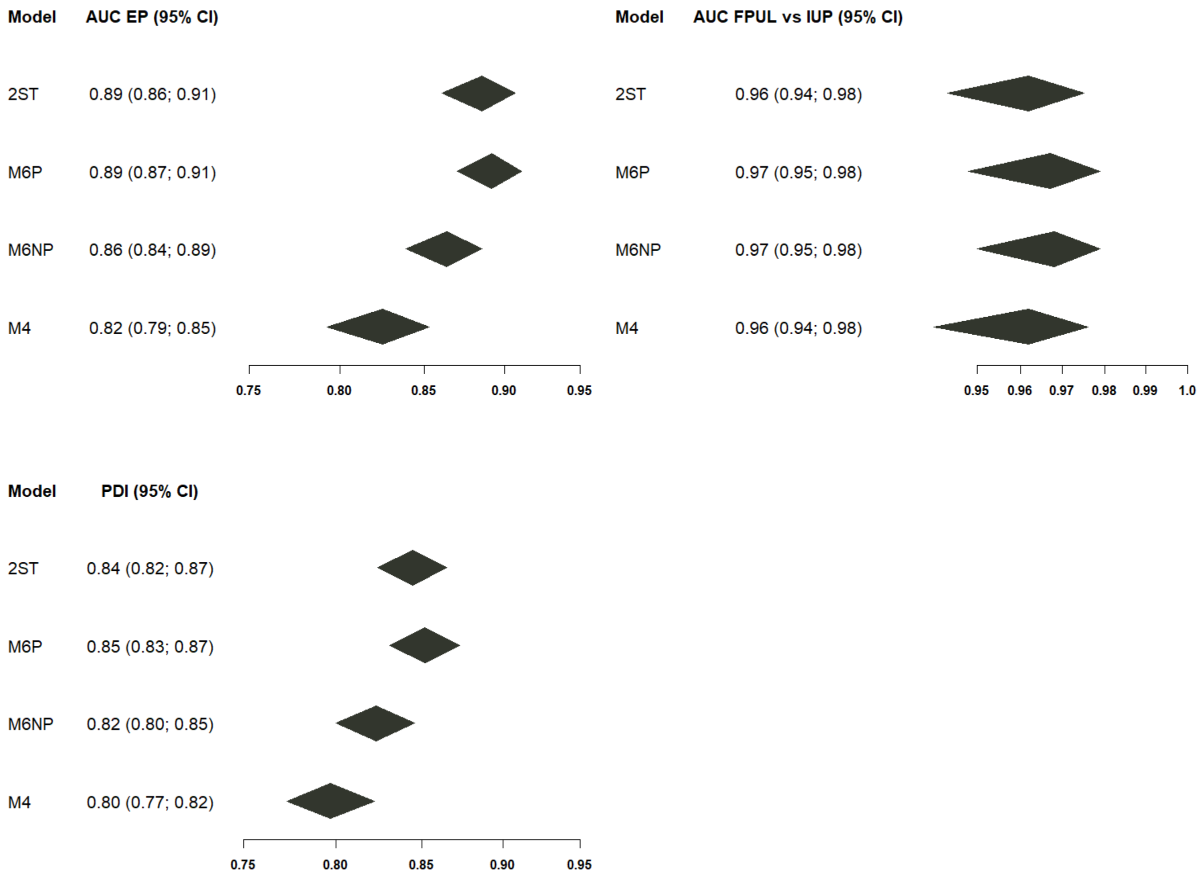

Figure S25. Summary forest plot of area under the receiver operating characteristic curve (AUC) for ectopic pregnancy (EP), the AUC for failed pregnancies of unknown location (FPUL) vs intra-uterine pregnancies (IUP), and the Polytomous Discrimination Index (PDI) when using second beta human chorionic gonadotropin (BhCG) levels between 1-3 calendar days after the first. The diamonds refer to the meta-analysis of centre-specific results.

CI, confidence interval.

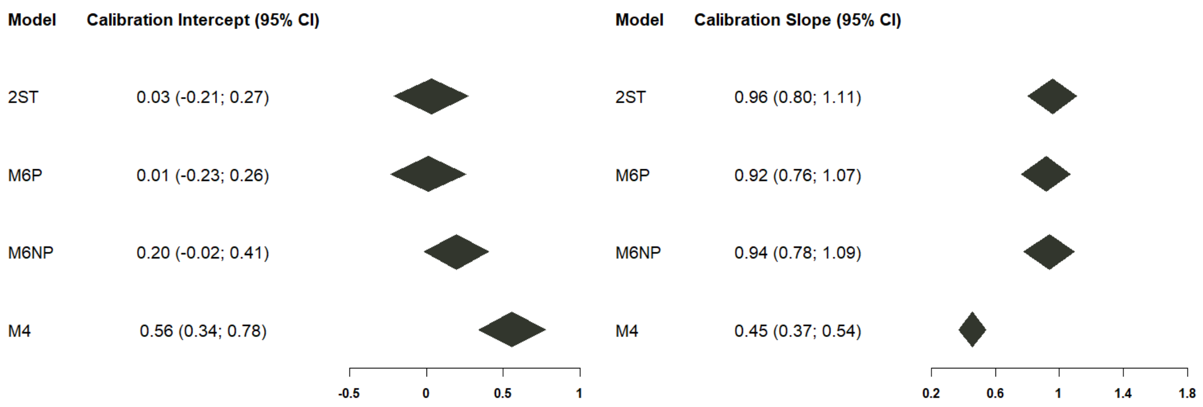

Figure S26. Summary forest plots of calibration intercept and calibration slope when using second beta human chorionic gonadotropin (BhCG) levels between 1-3 calendar days after the first. The diamonds refer to the meta-analysis of centre-specific results.
CI, confidence interval.

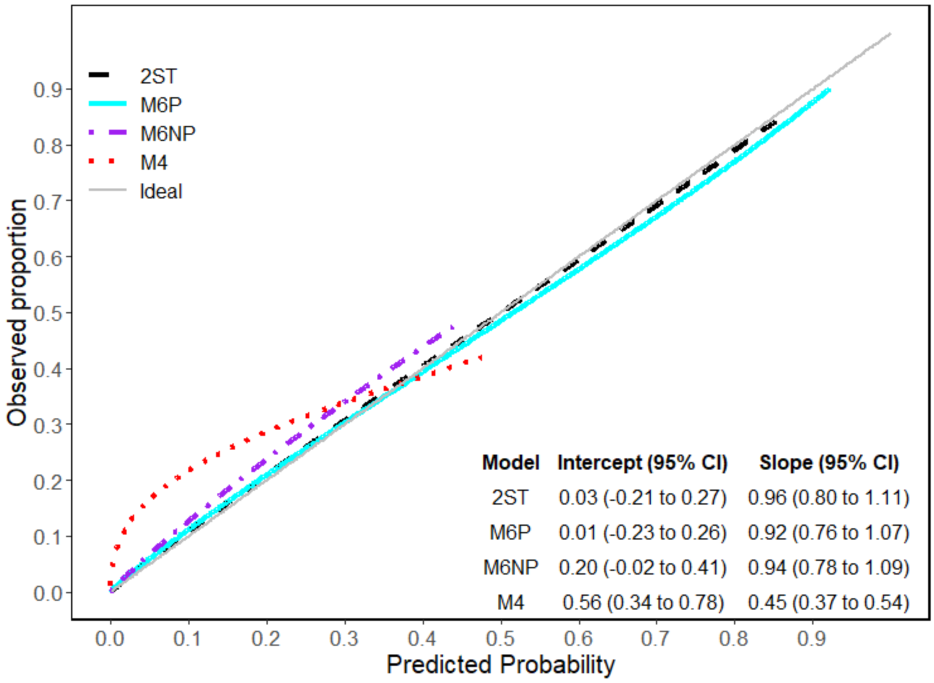

Figure S27. Summary calibration curves when using second beta human chorionic gonadotropin (BhCG) levels between 1-3 calendar days after the first.

CI, confidence interval.

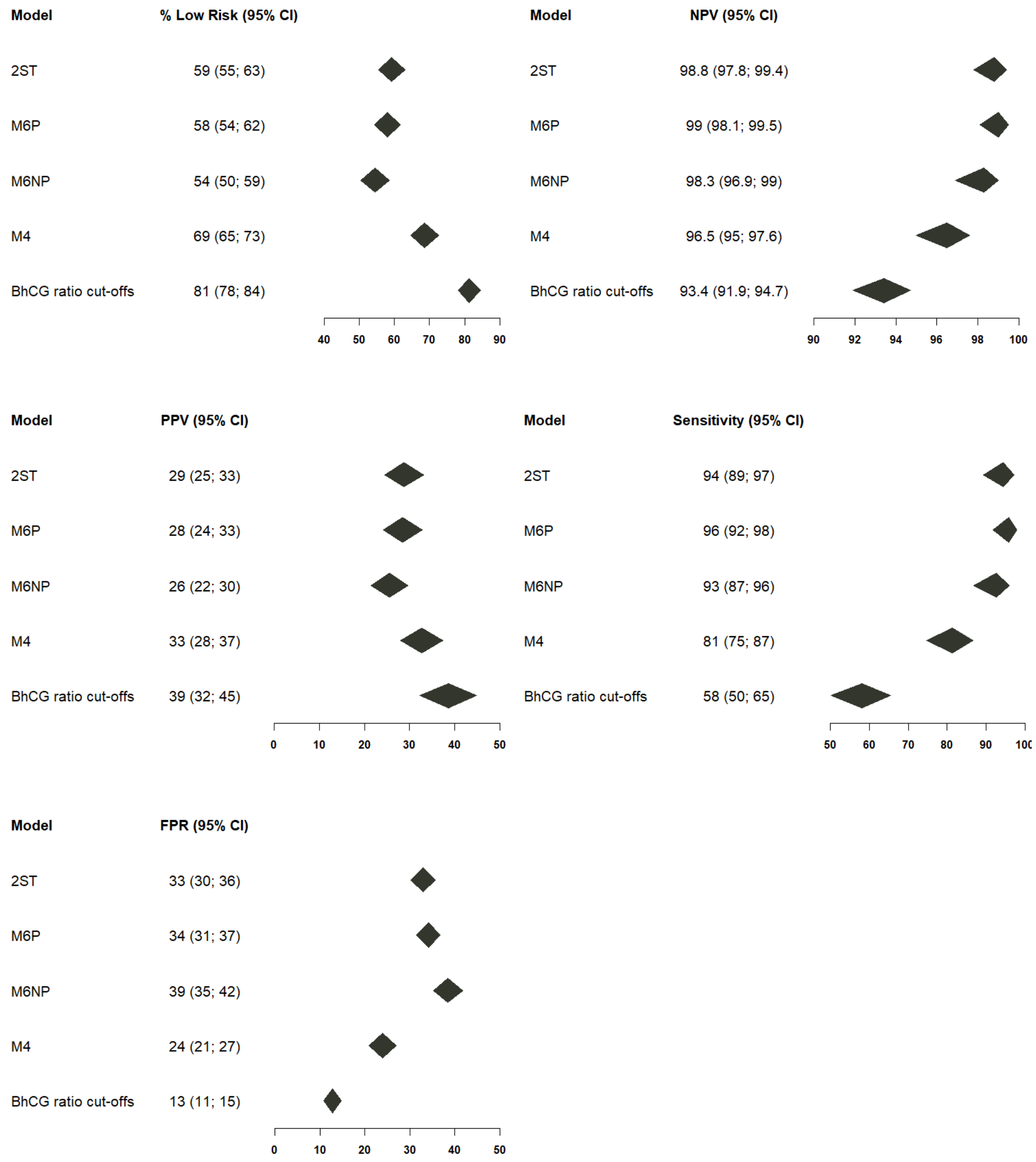

Figure S28. Summary forest plots of the percentage of patients classified as low risk, the negative predictive value (NPV), the positive predictive value (PPV), the sensitivity for ectopic pregnancy, and the false positive rate (FPR) when using second beta human chorionic gonadotropin (BhCG) levels between 1-3 calendar days after the first. The diamonds refer to the meta-analysis of centre-specific results.

CI, confidence interval.

iii. “As Treated” Analysis of 2ST

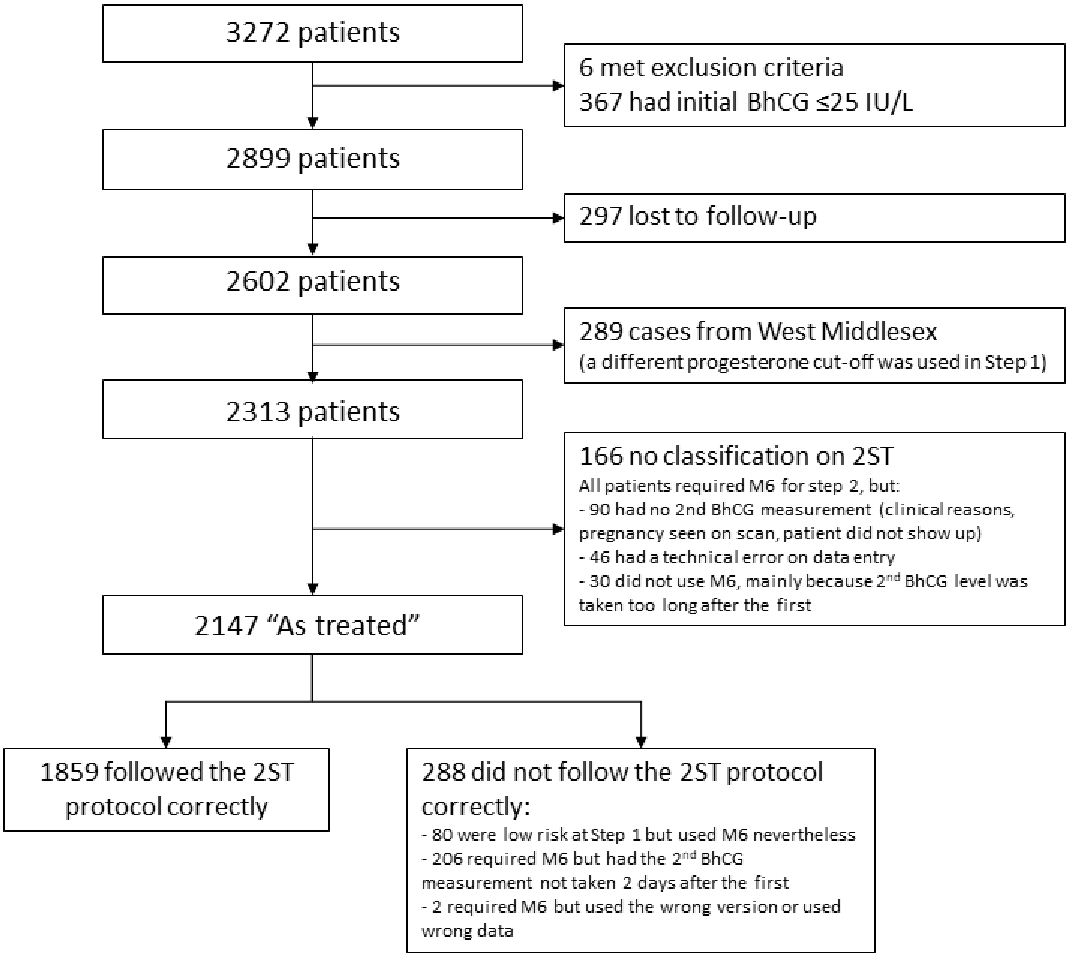

Figure S29. Flowchart of the “As treated” Analysis.

BhCG, beta human chorionic gonadotropin; FPUL, failed pregnancy of unknown location; EP, ectopic pregnancy; IUP, intra-uterine pregnancy.

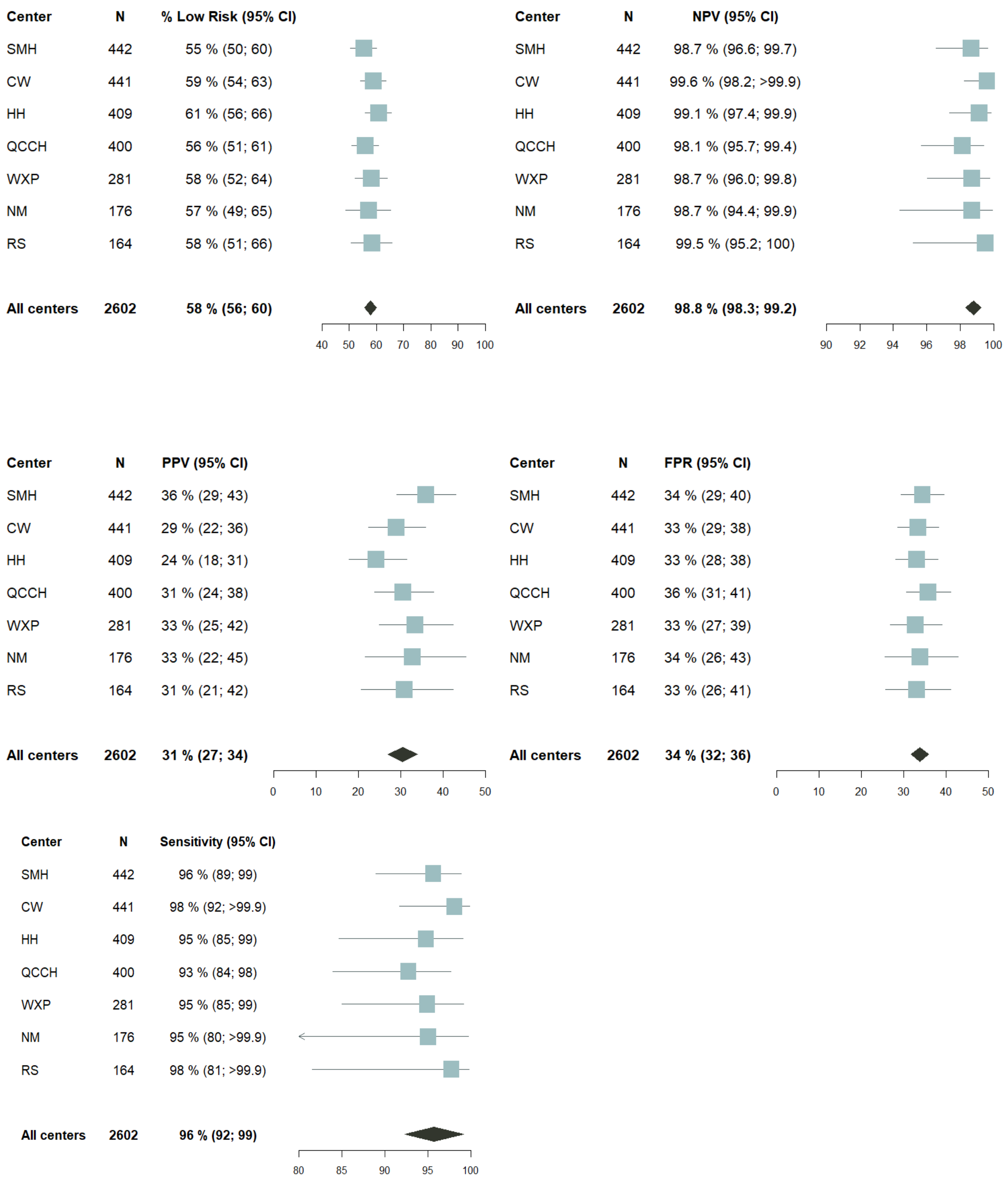

Figure S30. Centre-specific forest plots of the percentage of patients classified as low risk, the negative predictive value (NPV), the positive predictive value (PPV), the sensitivity for ectopic pregnancy, and the false positive rate (FPR) of the "As Treated" Analysis of 2ST. The diamonds refer to the meta-analysis of centre-specific results.

CI, confidence interval; SMH, St. Mary's; HH, Hillingdon; CW, Chelsea and Westminster; QCCH, Queen Charlotte and Chelsea; WXP, Wexham Park; NM, North Middlesex; RS, Royal Surrey.
