# Supplementary material for: External validation of models to predict the outcome of pregnancies of unknown location: a multicentre cohort study
Source: BJOG. 2020 Oct 7;128(3):552–62. doi: 10.1111/1471-0528.16497 (PMC7821217; doi:10.1111/1471-0528.16497)
Supplement: Supplementary file 2 — Table S1. Descriptive statistics of key variables by centre. Table S2. Interval between beta human chorionic gonadotropin (BhCG) measurements (in days). Table S3. Descriptive statistics in original model development dataset of M6NP, M6P and 2ST and the current external validation dataset. [file BJO-128-552-s002.pdf]

Table S1. Descriptive statistics of key variables by centre

Centre*	N	Age, years	Initial progesterone, nmol/L	Initial BhCG, IU/L	48h BhCG, IU/L[a]	BhCG ratio[a]	LFU	FPUL	IUP	EP/PPUL
SMH	508	32 (15-48)	12 (1-187)	527 (26-85000)	599 (8-30191)	1.01 (0.12-5.85)	66 (13%)	194 (38%)	176 (35%)	72 (14%)
CW	470	33 (16-50)	14 (1-333)	518 (26-1472)	608 (8-39520)	1.10 (0.10-4.00)	29 (6%)	224 (48%)	163 (35%)	54 (11%)
HH	472	30 (16-46)	8 (3-115)	540 (26-59757)	416 (8-109658)	0.59 (0.07-4.48)	63 (13%)	229 (49%)	136 (29%)	44 (9%)
QCCH	444	32 (14-48)	15 (1-219)	436 (26-105006)	537 (3-21154)	1.22 (0.11-4.25)	44 (10%)	176 (40%)	168 (38%)	56 (13%)
WMUH	299	32 (15-48)	5 (0.3-115)	431 (26-37406)	462 (5-7000)	0.75 (0.08-4.44)	10 (3%)	210 (70%)	63 (21%)	16 (5%)
WP	334	30 (16-45)	13 (0.3-179)	787 (27-126000)	593 (8-44103)	0.91 (0.01-6.20)	53 (16%)	129 (39%)	108 (32%)	44 (13%)
NM	186	31 (15-46)	11 (0.3-128)	422 (26-72550)	382 (5-17522)	0.80 (0.06-2.74)	10 (5%)	93 (50%)	56 (30%)	27 (15%)
RS	186	32 (16-45)	11 (1-123)	559 (30-20003)	569 (5-17454)	0.75 (0.01-5.91)	22 (12%)	83 (45%)	60 (32%)	21 (11%)
All	2899	32 (14-50)	11 (0.3-333)	519 (26-126000)	520 (3-109658)	0.90 (0.01-6.20)	297 (10%)	1338 (46%)	930 (32%)	334 (12%)

Statistics are shown as median (range).

[a] When a patient was on progesterone supplements, the progesterone value was considered a missing value.

[b] Second BhCG values not taken two days after the initial value were considered missing values, and were not included in the calculation.

Table S2. Interval between beta human chorionic gonadotropin (BhCG) measurements (in days)

Days	All women (N=2899)	Primary analysis (n=2602)	Lost to follow-up (n=297)
1	51 (2%)	45 (2%)	6 (3%)
2	2045 (86%)	1876 (86%)	169 (84%)
3	159 (7%)	148 (7%)	11 (5%)
4	55 (2%)	49 (2%)	6 (3%)
5-7	44 (2%)	36 (2%)	8 (4%)
8-14	17 (1%)	16 (1%)	1 (<1%)
>14	6 (<1%)	6 (<1%)	0
Missing values	10	10	0
No 2nd BhCG	512	416	96

Results are shown as n (%).

Table S3. Descriptive statistics in original model development dataset of M6NP, M6P, and 2ST and the current external validation dataset

	Development data [a]			External Validation (Current study; primary analysis population)		
**Variable**	**FPUL**	**IUP**	**EP/PPUL**	**FPUL**	**IUP**	**EP/PPUL**
N	785	501	163	1338	930	334
Age (years)						
Median (IQR)	32 (27-36)	30 (24-33)	32 (28-35)	32 (28-37)	31 (27-35)	32 (28-35)
Missing (n, %)	0	0	0	1 (<1%)	1 (<1%)	0
Initial progesterone (nmol/L)						
Median (IQR)	5 (3-10)	65 (47-85)	21 (11-39)	5 (2-9)	49 (30-69)	16 (8-29)
Missing (n, %)		*6% in total sample*		132 (10%)	91 (10%)	42 (13%)
Initial BhCG (IU/L)						
Median (IQR)	308 (104-975)	488 (253-887)	521 (200-1220)	362 (109-1458)	710 (329-1756)	480 (193-1038)
Missing (n, %)	0	0	0	0	0	0
Second BhCG (IU/L)						
Median (IQR)	143 (48-397)	1061 (515-1863)	604 (224-1266)	171 (54-565)	1260 (602-2820)	508 (230-1066)
Missing (n, %)		*4% in total sample*		481 (36%)	192 (21%)	53 (16%)
BhCG ratio						
Median (IQR)	0.37 (0.27-0.56)	2.14 (1.84-2.43)	1.20 (0.94-1.52)	0.36 (0.26-0.53)	2.06 (1.59-2.41)	1.19 (0.94-1.55)
Missing (n, %)		*4% in total sample*		481 (36%)	192 (21%)	53 (16%)

[a] The final M6 model was refitted on the combined development and validation data (n=2753), but the paper does not include descriptive statistics on this full sample. Regarding missing values, however, the paper only gives results for the full dataset of 2753 PUL. For progesterone, 177 (6%) missing values were reported, for second BhCG and BhCG ratio 109 (4%).

Legend: BhCG, beta human chorionic gonadotropin; UPT, Urine Pregnancy Test; US, ultrasound; FPUL, failed pregnancy of unknown location; EP, ectopic pregnancy; IUP, intra-uterine pregnancy; IQR, interquartile range.
