# Supplementary material for: External validation of models to predict the outcome of pregnancies of unknown location: a multicentre cohort study
Source: BJOG. 2020 Oct 7;128(3):552–62. doi: 10.1111/1471-0528.16497 (PMC7821217; doi:10.1111/1471-0528.16497)
Supplement: Supplementary file 3 — Appendix S1. Additional information on patient management based on 2ST. Appendix S2. Detailed information about the prediction models. Appendix S3. Imputation of missing data and statistical analysis. [file BJO-128-552-s003.pdf]

**Appendix S1.** Additional information on patient management based on 2ST.

There was a telephone follow-up service at each unit to contact each patient and provide reassurance and counselling as well as check the urine pregnancy test result. If the result was positive, women were advised to re-attend the EPAU for review. If the progesterone result was >2nmol/l, women were asked to re-attend 48 hours later for a repeat serum BhCG blood test. The two BhCG levels were entered onto the excel datasheet which contained an embedded algorithm for the M6 model which provided an instant risk prediction and recommended a management plan to the clinician using it (see figure 1). A version of M6 without progesterone as a predictor was used if the patient was on progesterone supplementation or if progesterone was not measured. Units were advised they did not have to measure serum progesterone levels if this was not part of their routine practice and they could move straight onto step 2. When using serum BhCG results, units were advised not to use the second BhCG result to obtain a risk prediction if it had been taken 8 hours before or after the 48 hour time point.

The M6 model classified a PUL case as 'high-risk' (likely diagnosis of EP/ PPUL) if the predicted risk of EP was ≥5%. If the risk was <5%, the predicted final outcome was either likely 'low-risk, probable FPUL' or 'low-risk, probable IUP' depending on which of these two outcomes had the highest likelihood based on M6. Women with a 'low-risk probable FPUL' were asked to perform a urine pregnancy test 2 weeks later to ensure the result was negative. If the test was positive, the patient was brought back for review with a senior clinician and a repeat serum BhCG and/or TVS. 'Low-risk probable IUP' cases were advised to have a repeat TVS to confirm the presence of an IUP after 1 week. Women with a PUL predicted to be 'high-risk, probable EP' were asked to attend the EPAU within 48 hours for senior clinical review and a repeat TVS (figure 1). If the pregnancy was still not seen, a repeat serum BhCG was taken and an individualized plan was made dependent on the clinical situation. At each visit, women were advised to seek medical advice promptly if they had any concerns or worsening of their symptoms. Clinicians were informed that the two-step protocol was a guide offering advice on how to manage a woman with a PUL and should not be followed if the clinical situation warranted a different approach. Clinicians were also told that the M6 model should be used for guidance on follow-up and should not be used to determine whether medical/ surgical intervention was necessary – this responsibility always rested with the managing clinician.

**Appendix S2.** Detailed information about the prediction models.

**1. The M4 risk prediction model**

The M4 risk prediction model was published in 2007.[1] It is a multinomial logistic regression model that uses the beta human chorionic gonadotropin (BhCG) ratio and the log of the average BhCG levels (in IU/L), i.e. the natural logarithm of (BhCG level at presentation + BhCG level at 48 hours)/2, as predictors. Its formulas are:

$$\log\left(\frac{P_{FPUL}}{P_{IUP}}\right) = 5.8803 - 5.5594 \times (BhCGratio - 1.17) + 2.0504 \times (BhCGratio - 1.17)^2$$

$$- 1.1795 \times \log(averageBhCG)$$

$$= z_1$$

$$\log\left(\frac{P_{EP}}{P_{IUP}}\right) = 0.3857 - 0.2627 \times (BhCGratio - 1.17) - 3.9319 \times (BhCGratio - 1.17)^2$$

$$- 0.0572 \times \log(averageBhCG)$$

$$= z_2$$

$P_{FPUL}$, $P_{IUP}$, and $P_{EP}$ represent the estimated risks of the three categories.

These risks are derived from the following formulas:

$$P_{FPUL} = \frac{e^{z_1}}{1 + e^{z_1} + e^{z_2}}$$

$$P_{IUP} = \frac{1}{1 + e^{z_1} + e^{z_2}}$$

$$P_{EP} = \frac{e^{z_2}}{1 + e^{z_1} + e^{z_2}}$$

EP, ectopic pregnancy; IUP, intrauterine pregnancy; FPUL, Failed Pregnancy of Unknown Location.

**2. The M6P and M6NP risk prediction models**

The M6 model was published in 2016.[2] It is also a multinomial logistic regression model. There is one version with progesterone as a predictor (M6P), and one version without (M6NP).

The linear predictors $z_1$ and $z_2$ for M6P are:

$$z_1 = log\left(\frac{P(FPUL)}{P(EP)}\right)$$

$$= 3.3265$$

$$-0.3477 \times log(initial\ hCG)$$

$$-0.4501 \times log(initial\ progesterone)$$

$$-5.6713 \times log(hCG\ ratio)$$

$$+1.0781 \times [log(hCG\ ratio)]^2$$

$$+1.0529 \times log(hCG\ ratio) \times log(initial\ progesterone)$$

$$z_2 = log\left(\frac{P(IUP)}{P(EP)}\right)$$

$$= -5.0661$$

$$+0.3813 \times log(initial\ hCG)$$

$$+0.5452 \times log(initial\ progesterone)$$

$$-5.2825 \times log(hCG\ ratio)$$

$$+1.3498 \times [log(hCG\ ratio)]^2$$

$$+2.1392 \times log(hCG\ ratio) \times log(initial\ progesterone)$$

The linear predictors $z_1$ and $z_2$ for M6NP are

$$z_1 = log\left(\frac{P(FPUL)}{P(EP)}\right)$$

$$= 2.5506$$

$$-0.4242 \times log(initial\ \mathrm{B}hCG)$$

$$-2.9502 \times log(BhCG\ ratio)$$

$$+2.1765 \times [log(BhCG\ ratio)]^2$$

$$z_2 = log\left(\frac{P(IUP)}{P(EP)}\right)$$

$$= -3.2842$$

$$+0.4072 \times log(initial\ \mathrm{B}hCG)$$

$$+1.9238 \times log(BhCG\ ratio)$$

$$+2.8952 \times [log(BhCG\ ratio)]^2$$

BhCG is measured in IU/L, progesterone in nmol/L. Probabilities are obtained in the same way as for M4.

**Appendix S3.** Imputation of missing data and statistical analysis.

1. Multiple imputation procedure

To validate the models, we have to address missing values in initial progesterone and beta human chorionic gonadotropin (BhCG) ratio. Also, some women were on progesterone supplements. For these women, we considered the progesterone value to be missing as well. Regarding BhCG ratio, a second BhCG measurement is required that should be based on blood sample taken 48 hours after the first. In practice, there was considerable variability in the timing of the second BhCG measurement. We decided to use second BhCG levels based on blood samples taken two calendar days after the first blood sample, and to consider other available second BhCG levels as missing. In our study, when a second BhCG level was available, it was taken two calendar days after the first in 86% of the cases (Table S2). More generally, because clinicians were recommended not to take a second BhCG level and use M6 when initial progesterone was ≤2 nmol/L, the study set-up created missing values in a specific subgroup of patients. Specifically, the design lead to a higher amount of missing values in pregnancies of unknown location (PUL) with a final outcome of failed PUL (FPUL). Finally, a group of women (10%) were lost to follow-up, such that their final PUL outcome was not known.

Women that were lost to follow up were included in the imputation process, but not in the primary analysis of this paper. This is the 'multiple imputation then deletion' method.[3] In a sensitivity analysis, we included these women using the imputations of their final outcome.

The imputation was done using fully conditional specification using the mice package in R.[4] In the imputation procedure, we included the final outcome, the biomarker values that are used as predictors in the model, values that are related to the final PUL outcome according to the literature, and other variables that could be related to the missingness of the biomarkers or the final outcome.[2,5] In specific, we included the following variables in the imputation procedure: centre (nominal; 8 levels), final PUL outcome (nominal; we used four levels for the imputation procedure: failed PUL, viable intra-uterine pregnancy (IUP), non-viable IUP, ectopic pregnancy (EP)/persisting PUL (PPUL)), log of the BhCG level at presentation, log of the BhCG level two days later, log of the initial progesterone level, patient age, level of vaginal bleeding (ordinal; 5 levels), history of EP (binary), and PUL status according to Barnhart's consensus statement (nominal; true PUL, probable miscarriage, probable IUP, probable EP).[6] 100 imputed datasets were generated.

In the imputation, we make a distinction between PUL that turn out to be viable IUP or non-viable IUP. The viability status may give additional information for generating imputations. However, for 95 IUP, the viability status was not recorded. Hence their final outcome was set to missing. As a result, the imputation procedure could impute any outcome (FPUL, viable IUP, non-viable IUP, or EP/PPUL) even though we know these cases are IUP. Therefore, when analysing the imputed datasets, the outcome of these cases was always set back to IUP.

Convergence plots of the imputed variables are shown in Figure A2.1. Distributions of observed and imputed values for initial progesterone second BhCG, and final PUL outcome are shown in Figure A2.2 and Table A2.1. The missing values, after imputation, were more often FPUL than IUP.

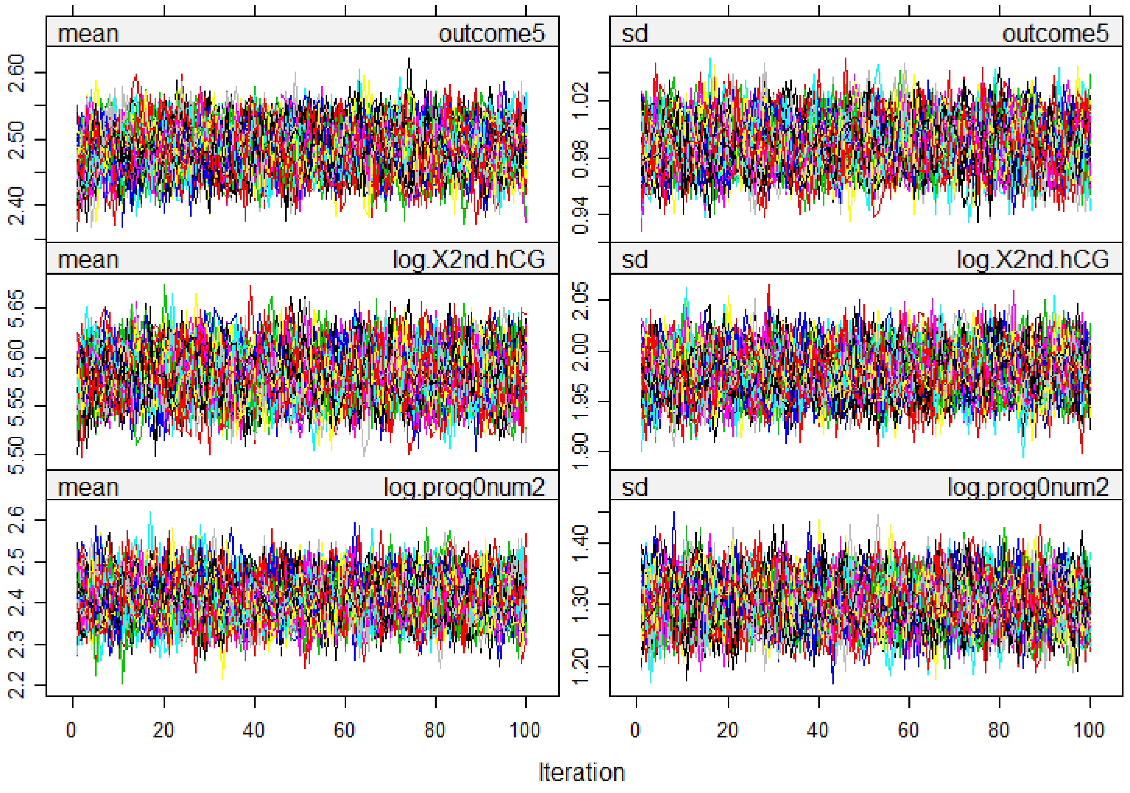


**Figure A3.1. Convergence plots.**

The plots refer to the imputations of the outcome variable (outcome5), log of the 2$^{nd}$ BhCG values (log.X2nd.hCG) and log of the initial progesterone (log.prog0num2) values. The plots on the left show the mean value, the plots on the right show the standard deviation (sd). The x-axis refers to the iteration (1 to 100). The coloured lines refer to the 100 imputations. The multinomial reference standard (outcome5) was used as a nominal variable in the imputations, despite it being represented as a numerical variable in these plots.

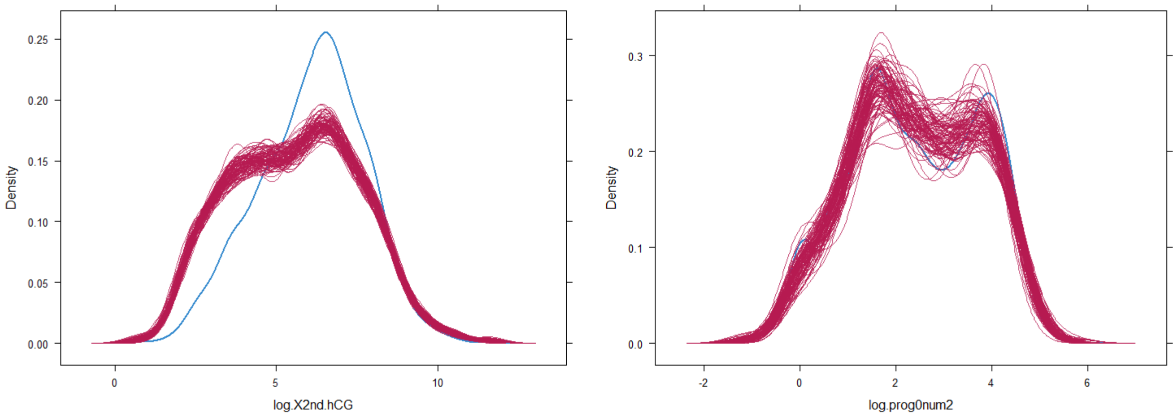


**Figure A3.2. Density plot for the log-transformed 2nd BhCG (log.X2nd.hCG) and initial progesterone (log.prog0num2) measurements.**
The blue curve is the density of the values that were observed (i.e. were not missing), the red curves are the densities for each of the 100 imputations of the values that were missing. The imputed values for both 2$^{nd}$ BhCG and progesterone were lower than the observed values, in particular for the 2$^{nd}$ BhCG level.

**Table A3.1. Distribution of final PUL outcome for observed and missing values after imputation.**

Lost to follow-up cases were imputed to be more often failed PUL than PUL with observed outcome.

| **Final PUL outcome** | **Observed** | **Imputed *** |
|---|---|---|
| Ectopic Pregnancy (EP) | 13.3% | 11.1% |
| Failed Pregnancy of Unknown Location (FPUL) | 53.4% | 64.7% |
| Intra-uterine Pregnancy (IUP) | 33.3% | 24.2% |

*Set of 297 observations that were Lost to Follow-up, hence final outcome was missing. Percentages were calculated on the pooled set of 100 imputations for these 297 observations.

2. Analysis of multiply imputed data

In this paper, % of PUL classified as low risk, negative predictive value (NPV), positive predictive value (PPV), sensitivity and false positive rate (FPR) are presented together with 95% confidence intervals. To obtain these results after multiple imputation, we implemented the following approach. In each of the 100 completed datasets after multiple imputation, % of low risk, NPV, sensitivity, FPR and their standard errors were calculated on the logit scale. For that, we used intercept only logistic regression. The results were combined across the completed datasets using Rubin's rules.[7] The

overall result is used to calculate the percentage and the 95% confidence interval on the original scale. Overall results across centres were obtained by combining centre-specific logit(proportions) and their standard error using random effects meta-analysis.

Of note, NPV and sensitivity were 100% in some, or even all, of the imputed datasets for a few centres. In such situations, the proportion and its standard error are undefined on logit scale. We addressed this issue by using a continuity correction to obtain a proportion below 100%. This was achieved by initially adding 0.5 to the number of events and to the number of non-events. The standard error of the logit of the continuity corrected proportion was estimated based on the approximate symmetry on the logit scale of the Wilson score confidence interval for proportions.[8] The estimated standard error is obtained as the difference between the logit of the lower confidence limit of the Wilson score confidence interval and logit of the proportion, divided by 1.96.

With respect to the area under the ROC curve (AUC), we obtained the logit(AUC) and its standard error. For this, the auc.nonpara.mw function of the auRoc package was used. In our primary analysis of multiply imputed data, the logit(AUC) values for each imputation were first combined using Rubin's rules per centre to get centre-specific results. 95% confidence intervals for logit(AUC) were calculated, and then the point estimate and the confidence limits were transformed to the original scale. An overall AUC was obtained by combining centre-level logit(AUC) and their standard error using random effects meta-analysis. 95% prediction intervals for the AUC for EP/PPUL were derived from the meta-analysis. The Polytomous Discrimination Index (PDI) and its estimated standard error for each imputation, were obtained with the use of the ests function in the mcca package in the primary analysis of multiply imputed data. Then, we used Rubin's rules to combine the PDI and their estimated standard error per centre in order to obtain centre-specific results. For an overall PDI we used random effects meta-analysis to combine centre-level PDI and their standard error.

Similarly, regarding calibration, we calculated centre-specific calibration intercept and slope for the risk prediction models, and then combined their values per centre using Rubin's rules for each imputed dataset in order to obtain centre-specific results. Overall calibration intercept and slope was obtained by combining their centre-level values using random effects meta-analysis. We then plotted centre-specific and overall logistic (i.e. non-flexible) calibration curves for the risk prediction models after fitting a logistic recalibration model with random intercept and random slope for the (J=8) centers[9]:

$$logit\left(\frac{P(Y=1)}{1-P(Y=1)}\right) = a + a_j + \beta X + b_j X \text{, where}$$

$$\begin{bmatrix} a_j \\ b_j \end{bmatrix} \sim N\left(\begin{bmatrix} 0 \\ 0 \end{bmatrix}, \begin{bmatrix} \tau_\alpha^2 & \tau_{\alpha b} \\ \tau_{\alpha b} & \tau_b^2 \end{bmatrix}\right)$$

For 2ST, M6P, M6NP and M4, X is the linear predictor, which equals the logit-transformation of the estimated risk of an EP. The overall calibration slope equals $\beta$. For the calibration intercept, the calibration slopes are set to 1, such that the model reduces to:

$$logit\left(\frac{P(Y=1)}{1-P(Y=1)}\right) = a' + a_j' + X, \text{ where } a_j' \sim N(0, \tau_{\alpha'}^2)$$

The overall calibration intercept equals $\alpha'$.
The overall calibration curve is based on the fitted model for the calibration slope where $a_j$ and $b_j$ are set to 0. The centre-specific calibration curve for centre j uses the same fitted model, but with the

estimates for $a_j$ and $b_j$. In case of multiple imputation, the fixed effects ($\alpha$, $\beta$ and $\alpha'$) were combined using Rubin's rules, and the centre-specific effects ($a_j$, $b_j$ and $a_j'$) were averaged.

All meta-analyses (including the calculation of prediction intervals), that were performed with the rma.uni function in the metafor package, made use of the Sidik-Jonkman estimator to estimate the between-study variance.[10] Prediction intervals for the AUC were obtained from the predict function in the metafor package.

With respect to clinical utility using decision curve analysis, we calculated Net Benefit (NB) for risk thresholds between 3% and 10% to decide which patients to monitor more closely because of a potential EP.[11] For each centre and threshold, we made an average 2x2 cross-tabulation over the 100 imputed datasets. The cross-tabulation contrasts outcome (EP vs FPUL/IUP) vs classification (risk<threshold vs risk≥threshold). This was used to calculate NB. Using Bayesian trivariate random-effects meta-analysis, the centre-specific NBs at a given threshold were combined into an overall estimate.[12] We used weak realistic priors for separate elements of the between-setting variance-covariance matrix: weak half-normal priors for variances (bounded by zero), weak Fisher priors for correlations and vague normal prior distributions for the remaining parameters. This analysis was performed using WinBugs (https://www.mrc-bsu.cam.ac.uk/software/bugs/the-bugs-project-winbugs/).

3. Sensitivity Analyses

For the sensitivity analyses, we calculated centre-specific and overall AUCs, overall calibration curves per model, and classification results.

The sensitivity analyses of: i) the Inclusion of PUL that were lost to follow-up and the ii) inclusion of second BhCG measurements between 1 to 3 calendar days after the 0h measurement, involved the use of multiply imputed data and therefore we applied Rubin's rules to combine centre-specific results per imputed dataset in the aforementioned manner. The analysis of 2ST 'as treated' did not involve imputed data.

All meta-analyses were performed with the techniques described above.

**References**


1. Condous G, Van Calster B, Kirk E, Haider Z, Timmerman D, Van Huffel S, et al. Prediction of ectopic pregnancy in women with a pregnancy of unknown location. Ultrasound Obstet Gynecol Off J Int Soc Ultrasound Obstet Gynecol. 2007;29(6):680–7.

2. Van Calster B, Bobdiwala S, Guha S, Van Hoorde K, Al-Memar M, Harvey R, Farren J, Kirk E, Condous G, Sur S, Stalder C. Managing pregnancy of unknown location based on initial serum progesterone and serial serum hCG levels: development and validation of a two-step triage protocol. Ultrasound in Obstetrics & Gynecology. 2016 Nov;48(5):642-9.

3. Von Hippel PT. 4. Regression with missing Ys: an improved strategy for analyzing multiply imputed data. Sociological Methodology. 2007 Aug;37(1):83-117.

4. Groothuis-Oudshoorn K, Van Buuren S. Mice: multivariate imputation by chained equations in R. J Stat Softw. 2011;45(3):1-67.

5. Van Calster B, Condous G, Kirk E, Bourne T, Timmerman D, Van Huffel S. An application of methods for the probabilistic three-class classification of pregnancies of unknown location. Artificial Intelligence in Medicine. 2009 Jun 1;46(2):139-54.

6. Barnhart K, Van Mello NM, Bourne T, Kirk E, Van Calster B, Bottomley C, et al. Pregnancy of unknown location: a consensus statement of nomenclature, definitions, and outcome. Fertil Steril. 2011;95(3):857–66.

7. Rubin DB. Multiple imputation for nonresponse in surveys. John Wiley & Sons; 2004 Jun 9.

8. Newcombe RG. Interval estimation for the difference between independent proportions: comparison of eleven methods. Statistics in medicine. 1998 Apr 30;17(8):873-90.

9. Wynants L, Vergouwe Y, Van Huffel S, Timmerman D, Van Calster B. Does ignoring clustering in multicenter data influence the performance of prediction models? A simulation study. Stat Methods Med Res. 2018;27(6):1723–36.

10. Viechtbauer W. Conducting meta-analyses in R with the metafor package. J Stat Softw. 2010;36(3):1–48.

11. Van Calster B, Wynants L, Verbeek JF, Verbakel JY, Christodoulou E, Vickers AJ, Roobol MJ, Steyerberg EW. Reporting and interpreting decision curve analysis: a guide for investigators. European urology. 2018 Dec 1;74(6):796-804.

12. Wynants L, Riley RD, Timmerman D, Van Calster B. Random-effects meta-analysis of the clinical utility of tests and prediction models. Stat Med. 2018;37(12):2034–52.
